# Electrophysiological heterogeneity in large populations of rabbit ventricular cardiomyocytes

**DOI:** 10.1093/cvr/cvab375

**Published:** 2022-01-10

**Authors:** Quentin Lachaud, Muhamad Hifzhudin Noor Aziz, Francis L Burton, Niall Macquaide, Rachel C Myles, Radostin D Simitev, Godfrey L Smith

**Affiliations:** Institute of Cardiovascular and Medical Sciences, University of Glasgow, Glasgow, UK; School of Mathematics and Statistics, University of Glasgow, Glasgow, UK; Institute of Mathematical Sciences, Faculty of Science, University of Malaya, Kuala Lumpur, Malaysia; Institute of Cardiovascular and Medical Sciences, University of Glasgow, Glasgow, UK; Institute of Cardiovascular and Medical Sciences, University of Glasgow, Glasgow, UK; School of Health and Life Sciences, Glasgow Caledonian University, Glasgow, UK; Institute of Cardiovascular and Medical Sciences, University of Glasgow, Glasgow, UK; School of Mathematics and Statistics, University of Glasgow, Glasgow, UK; Institute of Cardiovascular and Medical Sciences, University of Glasgow, Glasgow, UK

**Keywords:** Electrophysiology, Ventricle, Heterogeneity, Repolarization

## Abstract

**Aims:**

Cardiac electrophysiological heterogeneity includes: (i) regional differences in action potential (AP) waveform, (ii) AP waveform differences in cells isolated from a single region, (iii) variability of the contribution of individual ion currents in cells with similar AP durations (APDs). The aim of this study is to assess intra-regional AP waveform differences, to quantify the contribution of specific ion channels to the APD via drug responses and to generate a population of mathematical models to investigate the mechanisms underlying heterogeneity in rabbit ventricular cells.

**Methods and results:**

APD in ∼50 isolated cells from subregions of the LV free wall of rabbit hearts were measured using a voltage-sensitive dye. When stimulated at 2 Hz, average APD_90_ value in cells from the basal epicardial region was 254 ± 25 ms (mean ± standard deviation) in 17 hearts with a mean interquartile range (IQR) of 53 ± 17 ms. Endo-epicardial and apical-basal APD_90_ differences accounted for ∼10% of the IQR value. Highly variable changes in APD occurred after IK(r) or ICa(L) block that included a sub-population of cells (HR) with an exaggerated (hyper) response to IK(r) inhibition. A set of 4471 AP models matching the experimental APD_90_ distribution was generated from a larger population of models created by random variation of the maximum conductances (*G*_max_) of 8 key ion channels/exchangers/pumps. This set reproduced the pattern of cell-specific responses to ICa(L) and IK(r) block, including the HR sub-population. The models exhibited a wide range of *G*_max_ values with constrained relationships linking ICa(L) with IK(r), ICl, INCX, and INaK.

**Conclusion:**

Modelling the measured range of inter-cell APDs required a larger range of key *G*_max_ values indicating that ventricular tissue has considerable inter-cell variation in channel/pump/exchanger activity. AP morphology is retained by relationships linking specific ionic conductances. These interrelationships are necessary for stable repolarization despite large inter-cell variation of individual conductances and this explains the variable sensitivity to ion channel block.

## Graphical Abstract

**Graphical Abstract:**
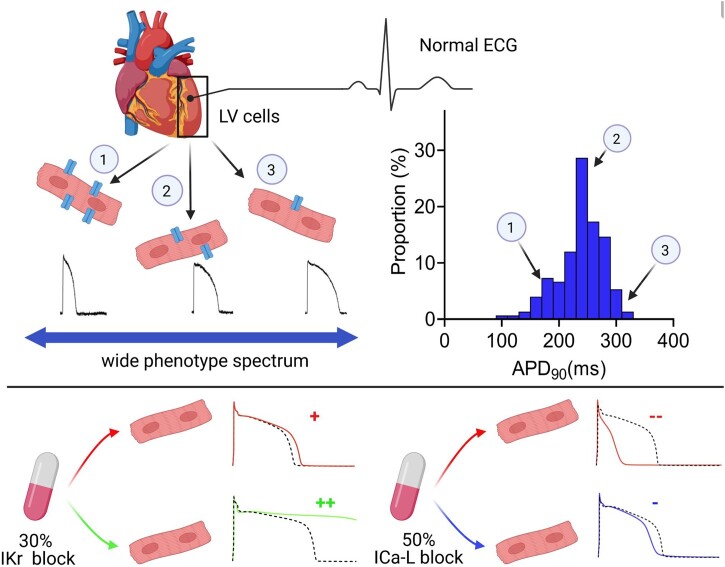

**Time for primary review: 37 days**

**This manuscript was handled by a Consulting Editor, Professor David A. Eisner.**

## 1. Introduction

The ventricular action potential (AP) waveform is the crucial first step in excitation–contraction (E-C) coupling. The time-course and magnitude of AP Phases 0–3 are a consequence of a complex pattern of trans-sarcolemmal current flow mediated by ion channels, electrogenic exchangers, and pumps. Any change to the dynamic balance of ionic currents caused by environmental (e.g. drugs) or genetic factors (e.g. single nucleotide polymorphisms) affects the AP waveform and can have fatal pro-arrhythmic consequences.^1^ The timing of repolarization in the left ventricle (LV), and hence the QT interval, is key to stable ventricular electrophysiology. The average corrected QT value (QT_c_) in humans is close to 400 ms [e.g. 408 ± 27 ms, mean ± standard deviation (SD)],^2^ with variation across a population quantified by an SD of 30–35 ms.^3^ Individuals with shorter or longer than normal QT_c_ values are at higher risk of death from cardiovascular causes.^1^ Genetic variation within the human population is thought to account for about 50% of the QT variation,^4^ presumably through variations in expression and activity/kinetics of ion channels associated with the AP. Computational models indicate that large variations in ion channel activity between individuals (inter-individual differences) may underlie the range of APD/QT times in the population.^5,6^ In support of this view, a recent study showed large inter-individual variation in mRNA levels for a range of ion channels and intracellular proteins.^7^ Computational studies have shown that this large inter-individual variation in ion channel activity can be tolerated through functional overlap and specific correlations between ion channels.^8,9^ This concept is established in neuroscience^10–12^ and evidence is now emerging of similar key relationships in cardiac-specific mRNA levels from molecular studies in humans.^7^

Both molecular and computational studies have treated the LV myocardium, a syncytium containing around 10^8^ myocytes, as having only regional variations in cellular AP waveform.^13^ A recent study^9^ matched a series of single-cell computational models with inter-subject and intra-subject variation in AP characteristics of human ventricular trabeculae (>10^3^ coupled myocytes).^14^ However, no studies to-date have assessed between-cell variation in AP waveform experimentally, although previous studies have demonstrated cell-to-cell variability in atrial^15,16^ and ventricular cells.^17^ In the past, technical limitations prevented large enough sample sizes (>30) being achieved in one heart, and therefore data represented combined inter- and intra-individual electrophysiological variability.

In this study, inter-cell variation in LV AP waveforms was examined within individual hearts and compared to regional differences and differences between individuals. The data revealed a large variation in APD, 2–3× that observed regionally within the intact ventricle. Furthermore, the addition of ion channel blocking drugs dofetilide and nifedipine caused a range of effects within cells from one animal suggesting that genetically identical cells contained sub-groups of cells with different responses to drugs. The mechanistic basis for this behaviour was examined using a previously published model of rabbit ventricular electrophysiology.^18^ This showed that the range of cellular electrophysiology within one individual could be explained by an even larger variation in the expression levels of key proteins if certain coupled expression relationships were retained. This population of models replicated the range and features of different drug responses and represents the first set of AP models to reproduce the underlying inter-cell variation in ventricular cells from individual hearts.

## 2. Methods

### 2.1 Cardiomyocyte isolation

Procedures were undertaken in accordance with the UK Animals (Scientific Procedures) Act 1986 under Project Licence (PPL IE143D87F). New Zealand White male rabbits (2–2.5 kg) were given an intravenous injection of 500 iU heparin together with an overdose of sodium pentobarbitone (100 mg/kg) and their hearts removed. Isolated hearts were perfused retrogradely (25 mL/min 37°C) with nominally Ca^2+^-free Krebs-Henseleit solution^19^ containing 0.6 mg/ml collagenase (Type 1, Worthington Chemical), 0.1 mg/mL protease (Type XIV, Sigma) for 6–8 min. The LV free wall was dissected, and the endocardial and epicardial layers were dissected out (1–1.5 mm layers). In some cases, basal and apical segments were segregated (∼1 cm separation).

Aliquots of cells were loaded with the voltage-sensitive dye FluoVolt (0.17 µL/mL, Thermo Fisher Scientific) in the Krebs medium for 25 min (20°C) before being washed with Kerbs-Henseleit solution (extracellular Ca^2+^ 1.8 mmol/L) as described previously^19^ and placed on a 35 mm dish with a cover-glass base in an environmentally controlled stage incubator (37 ± 1°C). The FluoVolt fluorescence signal was recorded from individual cells using a 40× (NA 0.95) objective lens. Excitation wavelength was 470 ± 20 nm using a light-emitting diode and emitted light was collected by a photomultiplier at 510–560 nm and digitized at 10 kHz. FluoVolt staining and AP signals were retained for up to 5 h The CellOPTIQ software controlled the XY position of the bath using a motorized stage, allowing cell locations to be saved for repeated measurements in the same cell, before and after drug. Uniform rod-shaped cells with apparent striations were stimulated at 2 Hz using a 2 ms duration voltage pulse at 1.5× threshold via carbon plate electrodes. Cells able to follow 2 Hz field stimulation for >4 min were included. This criterion may result in an underestimation of natural cell-to-cell variability, as shown by a recent study, ventricular cardiomyocytes stimulated at low rates (1 Hz) can exhibit very variable electrophysiology.^17^

### 2.2 *In silico* modelling of rabbit myocyte electrophysiology

A biophysically realistic mathematical model of rabbit ventricular cells^18^ describing ionic currents and Ca^2+^-handling in the rabbit ventricular myocyte was used. The model includes major transmembrane currents responsible for AP generation and incorporates multiple intracellular compartments for Ca^2+^ signalling. A preliminary parameter sensitivity analysis following the approach of Romero *et al.*^20^ indicated that the conductance of 8 ionic currents, namely sodium/potassium pump current (INaK), rapidly [IK(r)] and slowly activating inwardly rectifying potassium current [IK(s)], inward-rectifier current (IK1), transient outward potassium current (Ito), Na/Ca exchanger current (INaCa), L-type Ca current [ICa(L)] and background Chloride current (IClb), exert the largest influence on repolarization (see Supplementary material online, *Figure S12*). To capture inter-cell variability 30 000 variants with different values for these conductances were generated from uniform distributions using Latin hypercube sampling. The parameter ranges were partitioned in 30 000 equal size intervals, values were randomly sampled with one from each interval and were then randomly permuted giving an eight-dimensional, near-random sample of conductance values. Sampling every possible combination of parameter values in a high-dimensional space is computationally demanding, if not infeasible for all but the lowest resolutions. The Latin hypercube sampling method provides an efficient and unbiased coverage of complicated parameter spaces with a user control of the number of samples required. The Latin hypercube sampling method has been used extensively since the 1970s and is popular in the context of constructing experimentally calibrated populations of cardiac models.^9,21–23^ Each model in the random initial population was integrated in time with a relative tolerance of 0.2 × 10^−6^ employing an adaptive-step, adaptive-order method for systems of stiff ordinary differential equations based on numerical differentiation formulas,^24^ as implemented in MATLAB (Version 9.6 R2019a Natick, MA, USA: The MathWorks Inc). Various re-parametrizations of the original Shannon model,^18^ are available in the literature.^25^ We do not expect significant advantages in using a different parametrization. In fact, such a parametrization is not essentially different from any member of the randomized populations considered in our study as it retains the exact same mathematical form and structure of the model equations and only differs in its parameter values. Each member of the initial population was stimulated by a constant current pulse at 2 Hz, a train of 1000 APs was computed, and the last two APs were recorded for depolarization/repolarization ‘biomarker’^9^ extraction and further analysis. Model variants were rejected from the initial sample population if they failed to depolarize above 0 mV or if they had a resting *V*_m_ >−65 mV. Model variants were also removed if the difference in APD90 between the last two APs was >5 ms (∼3% of APD90), for further details see subsection ‘Stability of the action potential train’ in the Supplementary material online, *Methods*. Traditionally, modelling studies have focussed on a single model taken as a typical representation and used to explain experimental results, elucidate mechanisms and generate predictions. More recently, population-based modelling approaches have become popular and have been applied to perform (i) parameter sensitivity analysis, including mainly maximal ionic conductances but also parameters controlling rates of ion transport, for example, gate kinetics and voltage dependances^26,27^ and (ii) to explore patterns of variability between behavioural classes,^8,28,29^ e.g. normal response vs. hyper-response (HR). The main focus of these studies is to compare populations of APs generated by experimental and computational methods with the simplest possible AP repolarization biomarker (APD_90_), therefore further analysis of the population of computational models^26,30^ was reserved for future studies.

### 2.3 Statistical analyses

These were carried out using GraphPad Prism 8 and the statistical computing environment R. Comparisons between two normally distributed groups were made using Student’s *t*-tests, paired where appropriate. Non-normally distributed, unpaired groups were compared using the non-parametric Mann–Whitney test, and Wilcoxon matched-pairs signed-rank test for paired sets. Comparisons between three or more groups were made using one-way analysis of variance, repeated-measures where appropriate, with Bonferroni’s correction for multiple comparisons. A value of *P* < 0.05 was considered statistically significant. Relationships between pairs of ionic current conductances were analysed using a Spearman’s rank partial correlation coefficient to eliminate effects of other simultaneously varying conductances and to limit the sensitivity to outliers.

## 3. Results

### 3.1 Distribution of APD values from a sample of collocated rabbit ventricular cells

**Figure 1:**
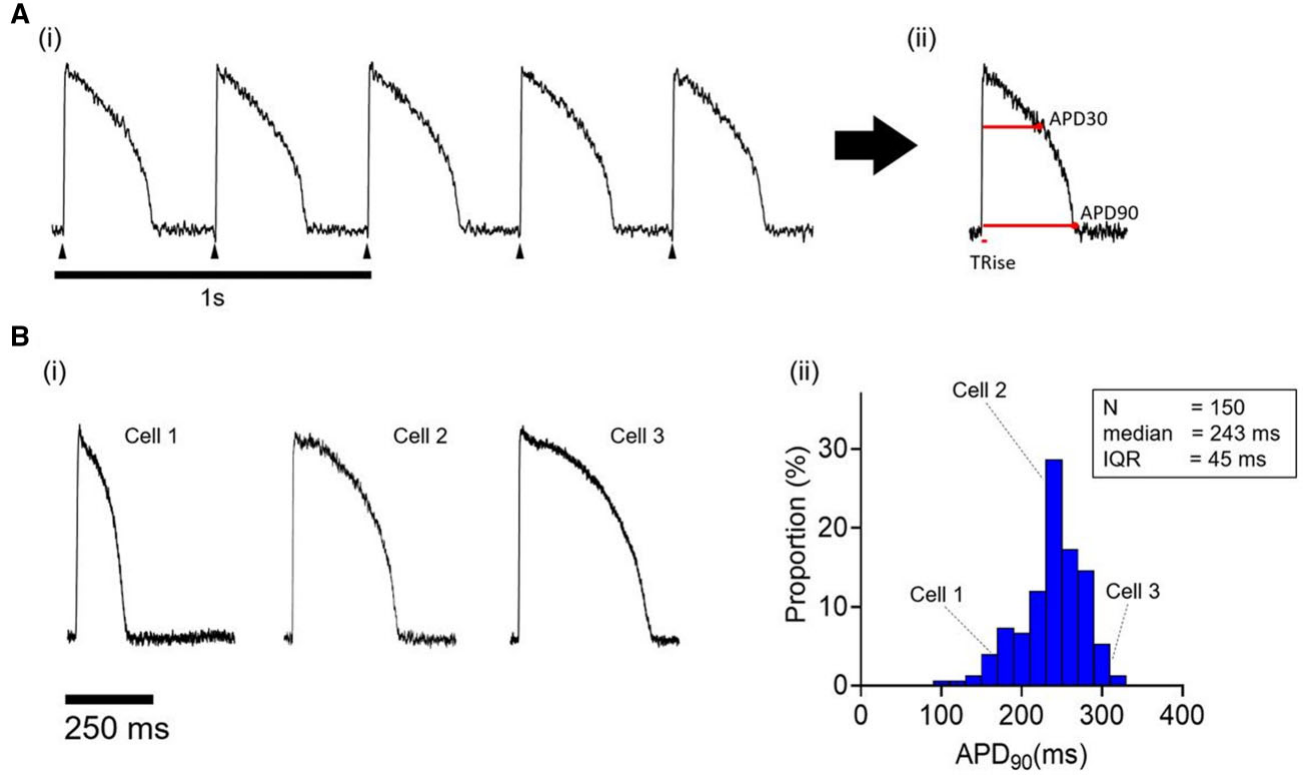
[*A(i)*] Optical APs recorded during field stimulation of a single isolated rabbit cardiomyocyte. [*A(ii)*] Averaged AP of train in [*A(i)*], including TRise, time from 10% to 90% of upstroke; APD_30_, time from mid-point of upstroke to 30% repolarization; APD_90_, time from mid-point of upstroke to 90% repolarization. [*B(i)*] APs sampled from three cardiomyocytes from a single rabbit LV. [*B(ii)*] APD_90_ distribution in 150 cells from the same LV.

As shown in *Figure 1A*, brief (∼2.5 s) recordings of optical APs were acquired in rapid succession from >100 single rabbit LV cardiomyocytes after stimulation for 4–5 min at 2 Hz. The beat-to-beat variability of APD_90_ was assessed as described previously^16^ and was <2% at 2 Hz (Supplementary material online, *Figure S1*), consistent with micro-electrode recordings.^31^

AP biomarkers were derived from the averaged waveform [*Figure 1A(ii)*]. Three example waveforms of averaged optical AP signals are shown in *Figure 1B(i)* to indicate the range of APDs encountered from contracting cells of normal morphology selected at random in the dish. The distribution of APD_90_ values from 150 cells recorded in a single session from a single heart is shown in *Figure 1B(ii)*. A skewed distribution is observed with a median APD_90_ of 243 ms and an interquartile range (IQR) of 45 ms. The distribution, median, and IQR values of other AP parameters are shown in Supplementary material online, *Figure S2*. Similar AP characteristics were recorded from LV myocytes from 17 hearts, and IQRs were independent of dissociation yield (data not shown). The APD_90_ median and IQR were dependent on stimulation frequency (Supplementary material online, *Table S1*). The IQRs at 1 Hz were ∼100% >2 Hz (84 ± 12 vs. 38 ± 4 ms, *P* < 0.01) and values at 3 Hz stimulation (27 ± 4 ms) are ∼30% smaller than those at 2 Hz (*P* < 0.01).

### 3.2 Distribution of APD values in subregions of the LV free wall and between different hearts

**Figure 2:**
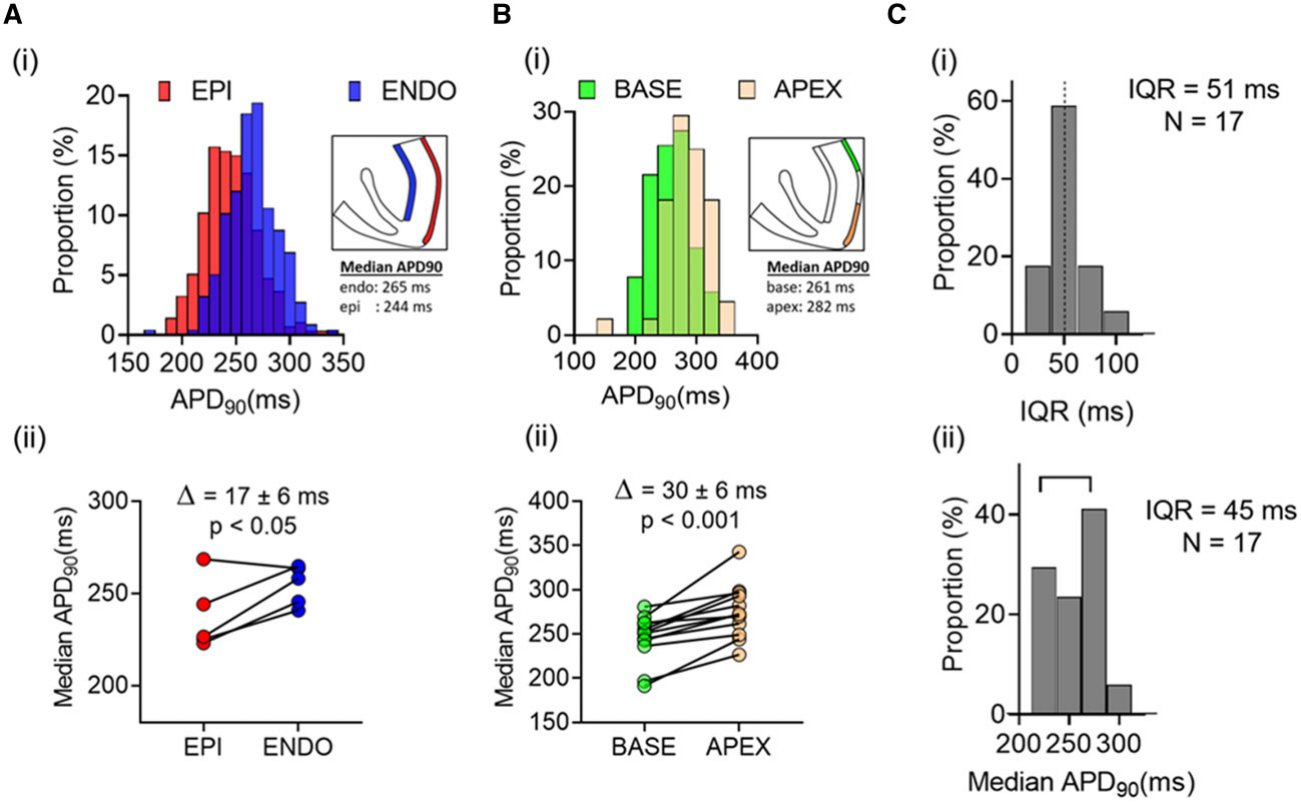
[*A(i)*] Distributions of APD_90_ in LV endocardial and epicardial cells from a single heart. [*A(ii)*] Comparison of median endocardial and epicardial APD_90_ in five hearts. [*B(i)*] Distributions of APD_90_ in LV basal and apical cells from a single heart. [*B(ii)*] Comparison of median apical and basal APD_90_ in 12 hearts. [*C(i)*] Distribution of IQRs for APD_90_ in 17 hearts. [*C(ii)*] Distribution of median APD_90_ from basal LV epicardium in 17 hearts. Statistical comparison between the paired data sets shown in [*A(ii)*] and [*B(ii)*] was made using a Student’s *t*-test.

The large variation in APD_90_ at near-normal heart rates (120 bpm) includes apical/basal and endo/mid/epicardial myocytes that have different average APD_90_ as previously reported.^17–20^ To assess the contribution of regional heterogeneity to the overall range of APD_90_ values, relevant subregions were compared. As shown in *Figure 2A and B*, the distributions of APD_90_ from these subregions have similar spread with IQR ∼50 ms [*Figure 2C(i)*]. As expected, endocardial APD_90_ was longer than epicardial [17 ± 6 ms, *P* < 0.05; *Figure 2A(ii)*] and apical APD_90_ was longer than basal [30 ± 6 ms, *P* < 0.05; *Figure 2B(ii)*]. The relatively large inter-cell variation in APD_90_ compared with these regional differences means that most of the cells in different LV regions have overlapping electrophysiological properties. The mean AP data from example individual hearts is shown in Supplementary material online, *Tables S5* and *S6*.

To assess the population variation in APD_90_ and IQR from the same region, median and IQR of basal epicardial cells were compared across 17 hearts at 2 Hz. *Figure 2C* shows that the average *intra*-heart IQR of 51 ms is comparable with the estimated *inter*-heart IQR of 45 ms (*n* = 17). Although the occurrence of inter-heart variation in APD_90_ is established, this is the first report to quantify intra-heart APD_90_ variability; and shows that, at 2 Hz stimulation, 50% of cells have APD_90_ >±25 ms from the median.

### 3.3 Effects of dofetilide and nifedipine on APD_90_ in populations of LV cells

**Figure 3:**
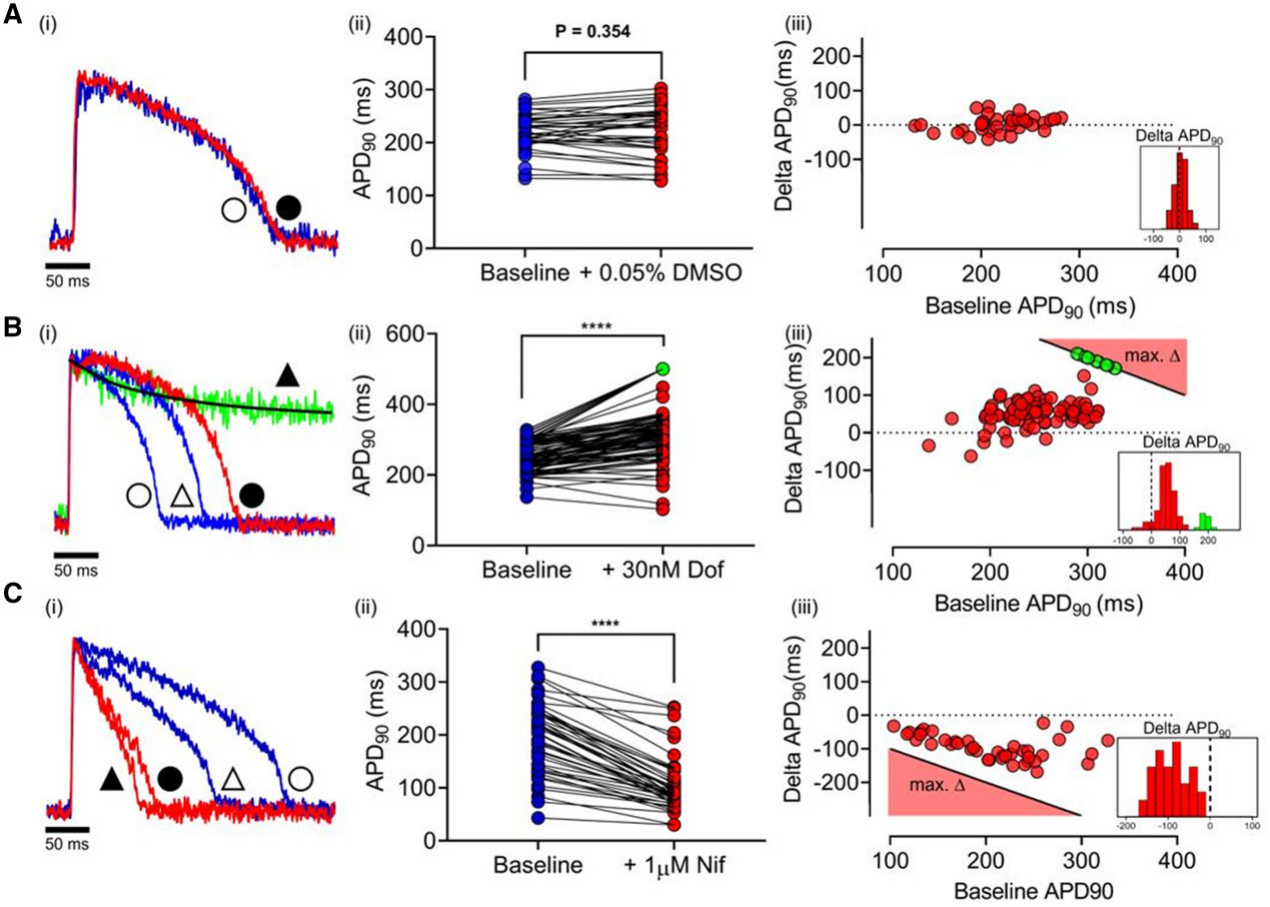
[*A(i)*] Single-cell AP traces at baseline (blue) and with vehicle (0.05% DMSO; red). [*A(ii)*] Paired APD_90_ before and after vehicle (*n* = 37 cells, 1 heart). [*A(iii)*] ΔAPD_90_ on addition of vehicle vs. baseline. [*B(i)*] Single-cell AP traces showing two typical NRs to 30 nM dofetilide. Baseline APs (blue, triangles) and after dofetilide (red, squares). Green trace shows typical HR. Symbols indicate pairing (unfilled = baseline; filled = drug). [*B(ii)*] Paired APD_90_ before and after dofetilide (*n* = 84 cells, 1 heart). [*B(iii)*] ΔAPD90 with dofetilide vs. baseline APD90. For HR cells the minimum ΔAPD90 is shown in green. The black line in [*A(iii)*], [*B(iii)*] denotes the minimum ΔAPD90 possible (e.g. a 280 ms AP which shows an HR response during 500 ms pacing must have prolonged by at least 220 ms (500–280 ms). Consequently, the equation of the black line is defined as: max ΔAPD90 = 500 − APD90. [*C(i)*] APs from two cells at baseline (blue) and following 1 µM nifedipine (red). [*C(ii)*] Paired APD90 before and after nifedipine (n = 44 cells, 1 heart). [*C(iii)*] ΔAPD90 with nifedipine vs. baseline APD90. The black line in [*C(iii)*] denotes the maximum ΔAPD90 possible (e.g. a 280 ms AP has a maximum ΔAPD90 of −280 ms). Consequently, the equation of the black line is defined as: max ΔAPD90 = − APD90. Dashed lines in histograms indicate ΔAPD90 = 0. Statistical comparison between the paired data sets shown in [*A(ii)*], [*B(ii)*], and [*C(ii)*] was made using the Wilcoxon matched-pairs signed-rank test; ****P < 0.001.

To examine the response to ion channel blocking drugs, APs were recorded before and after 30 nM dofetilide (*Figure 3B*) or 1 µM nifedipine (*Figure 3C*). Maximal concentrations were not used to minimize non-specific effects of the drugs^32^ or a high incidence of non-repolarizing responses. As shown in *Figure 3A*, dimethlysulphoxide (DMSO) control (0.05%) did not change APD_90_. ΔAPD_90_ was not correlated with baseline APD_90_ and was normally distributed (0 ± 23 ms mean ± SD).

*Figure 3B(i)* shows the response to dofetilide in two different cells with comparable baseline APD_90_. In one, APD_90_ increased, while in the other, dofetilide caused a prolonged plateau that did not repolarize within the cycle time (500 ms). The latter response was seen in 12% of cells (Supplementary material online, *Table S2*); termed ‘HR’. The range of APD_90_ values before and after addition of dofetilide from a representative heart is shown in *Figure 3B(ii)*, indicating most of the cells showed an increase in APD_90_ (‘normal responders’ NR), including some cells with no change (<5 ms) or even a decrease (10–20 ms) in APD_90_. This was comparable with the APD shortening seen in DMSO and so consistent with the normal temporal variation in repeated measures. The range of ΔAPD_90_ with dofetilide is shown in *Figure 3B(iii)*. In HRs APD_90_ quantification was not possible as, by definition the AP had not repolarized within the pacing cycle length, and so the minimum ΔAPD_90_ was plotted [e.g. a 280 ms AP which shows an HR response during 500 ms pacing must have prolonged by at least 220 ms (500–280 ms)]. Baseline APD_90_ was not significantly different between HR and NR, for the heart featured in *Figure 3*, NR vs. HR, 258 ± 48 vs. 299 ± 44 ms, (mean ± SD), indicating a considerable overlap of cells with similar APD_90_ values but distinct responses to IK(r) inhibition. The response to dofetilide in populations of cardiomyocytes from several hearts is summarized in Supplementary material online, *Table S2* (656 cells from 12 hearts). On average, 12% of cells in each heart were HRs, but in no cells were early after-depolarizations (EADs) recorded, suggesting an incidence of <2%.

*Figure 3C* shows the response of a population of cells from a single heart to 1 µM nifedipine. *Figure 3C(i)* illustrates the response of two cells in the same dish; one cell showed a pronounced decrease in APD_90_, the other a much smaller effect. The range of paired APD_90_ values are shown for a group of 44 cells from a single heart, most showed a decrease in APD_90_, but a sizeable group of cells failed to show a significant APD_90_ decrease (<5 ms). The relationship between baseline APD_90_ and ΔAPD_90_ is shown in *Figure 3C(iii)*. In general, cells with relatively short baseline APD_90_ showed minimal responses to nifedipine. Cells with longer APD_90_ values show a range of responses to nifedipine at the same concentration. The data are summarized in Supplementary material online, *Table S2*.

### 3.4 *In silico* modelling of experimentally observed APD_90_ values

**Figure 4:**
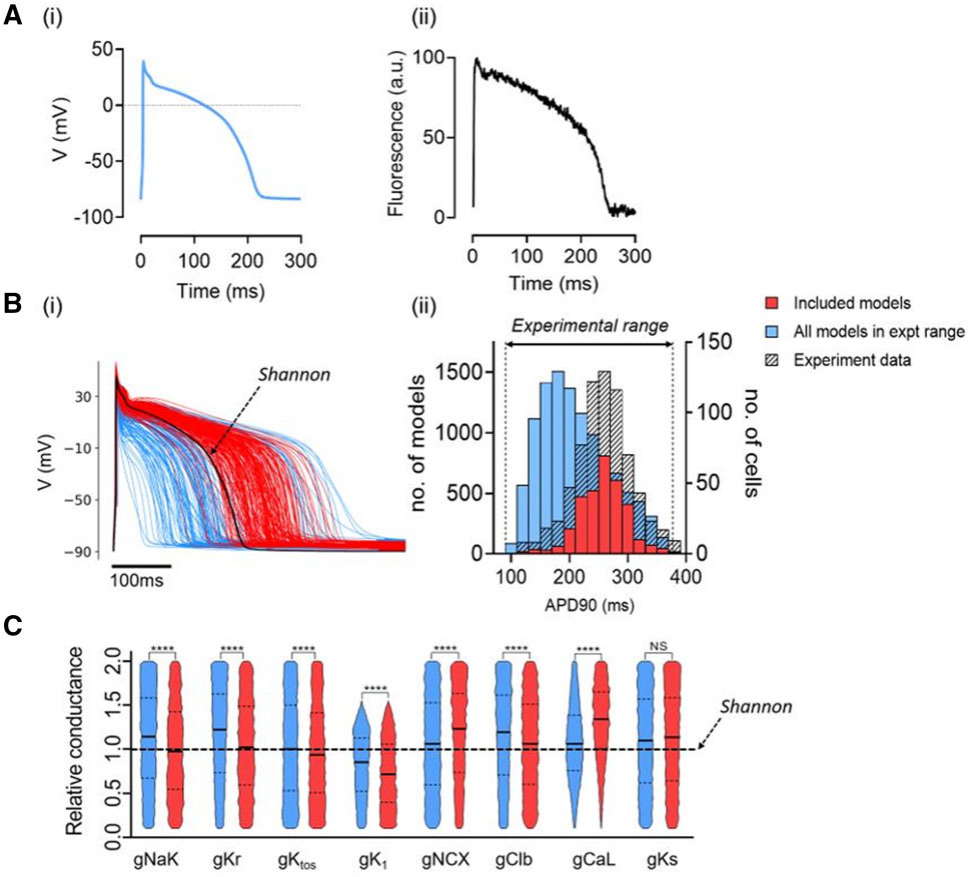
[*A(i)*] Calculated steady-state AP using Shannon model. [*A(ii)*] Optical AP; both stimulated at 2 Hz. [*B(i)*] APs from a population of models with channel conductances randomly selected from uniform distributions in a range of 10–200% of the standard values.^18^ APs with APD_90_ values within the experimental range are shown in blue and a further calibrated subset that matches the experimental APD value distribution is shown in red. Standard Shannon AP shown in black. [*B(ii)*] Histograms of APD_90_ in populations of Shannon models calibrated to experimental range (left axis, blue bars), to experimental range and distribution (left axis, red bars) and of populations of cells (right axis, hashed bars). (*C*) Violin plot of ion channel conductances between original (blue) and experimentally calibrated Shannon models (red). Statistical comparison between the unpaired data sets shown in (*C*) was made using the Mann–Whitney test (****P < 0.001).

To investigate the basis for this inter-cell variability in APD, an *in silico* pipeline was constructed using a well-established rabbit ventricular AP model.^18^ The model predicts a similar AP shape to those recorded experimentally but with a duration ∼50 ms lower at 2 Hz (*Figure 4A*). The maximum conductance of 8 ion channel/exchangers in the model was randomly varied between 10% and 200% of the original values to create 30 000 models (Supplementary material online, *Figure S3*). A two-step protocol was then used to calibrate the model population to experimental measurements. First, model variants for which APD_90_, APD_30_, or TRise (time from 10% to 90% of upstroke amplitude) were not in the respective experimental range were eliminated,^9^ leaving a population of 10 324 viable AP models (Supplementary material online, *Figure S3*). As shown in *Figure 4B(ii)*, APD_90_ values in this model population differed from those measured experimentally. Second, the range of model APD_90_ values was binned (*n* = 15) and model variants were removed at random from each bin until the model and experimental histograms were identical, an approach similar to that of.^33^ The final experimentally calibrated population included 4471 model variants with a median and IQR for APD_90_ that matched experimental values [*Figure 4B(ii)*]. *Figure 4C* shows the distributions of scaled conductance values for the viable AP models and the subset of experimentally-calibrated models. The experimentally calibrated models had lower mean Na/K pump, IK(r), IK(to) I(Clb), and IK(1) and higher INCX and ICa(L) conductances. The ranges of conductance for each ion/channel/exchanger/pump in the experimentally calibrated models were similar (10–200%) apart from IK(1) (max. ∼150%). As shown in Supplementary material online, *Figure S3*, the experimentally calibrated models show biologically similar AP waveform metrics to those derived experimentally.

Thus, *in silico* modelling of inter-cell differences in ion channel maximum conductance yielded a group of single-cell models that recapitulates the experimentally measured variability in APD_90_ and suggests that this variability can be accounted for by inter-cell variation in relative ion channel/pump/exchanger expression. Surprisingly, the variation in conductance values required to simulate the distribution was large compared to that of the resultant AP characteristics. Using %IQR coefficient (IQR/median × 100) as an index of variability, variability of APD_90_ is ∼25% at 2 Hz, whereas average conductance variability is ∼80% (Supplementary material online, *Table S3*).

There were distinct relationships between specific conductances in the experimentally calibrated models, the largest Spearman’s correlation coefficients (SCCs) were between ICa(L) vs. IK(r) and ICa(L) vs. I-Clb (SCC = 0.6), ICa(L) vs. INCX (SCC = −0.5) and ICa(L) vs. INaK [SCC = 0.5; Supplementary material online, *Figure S3C(iv)*]. This indicates that the observed APD variability can be generated by much larger variability in ion channel/exchanger/pump expression providing key relationships between these conductances are retained.

### 3.5 *In silico* modelling of dofetilide and nifedipine response using a population of AP models

**Figure 5:**
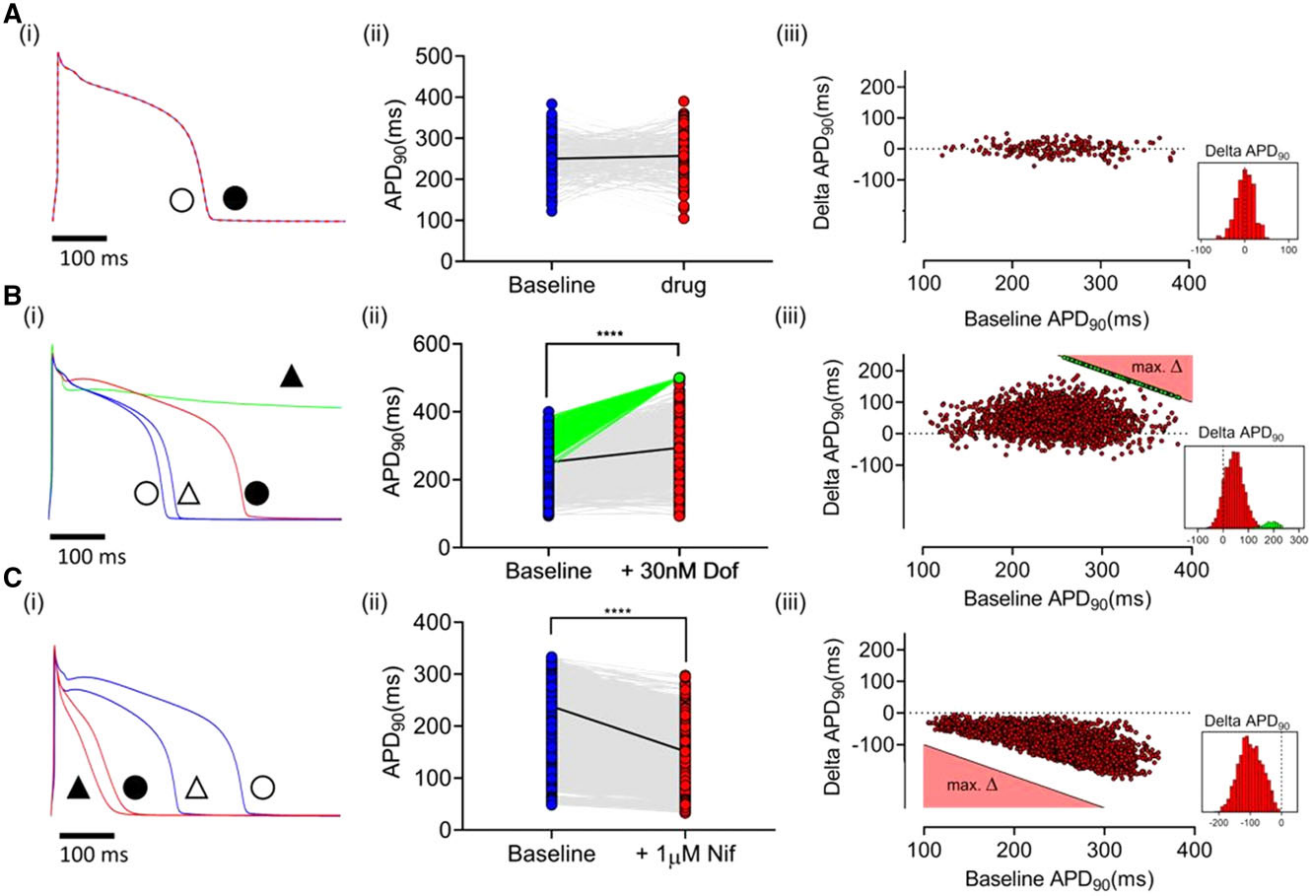
[*A(i)*] Model AP at baseline (blue) and post-vehicle (dashed red). [*A(ii)*] Dot-plot comparison of populations of model APs at baseline and following experimentally derived random effect. Individual cell responses (grey lines) and median behaviour (black line) shown. [*A(iii)*] Scatter plot of net random APD_90_ effect applied to model AP population. [*B(i)*] Two model APs at baseline (blue) and following IKr block. [*B(ii)*] Individual cell responses (grey), minimum HR (green), and median response (back) are shown. [*B(iii)*] ΔAPD in individual AP models (red). Minimum theoretical change observed in hyper-responders (cycle length—baseline APD) shown in green. [*C(i)*] Two models with different baseline APD (blue) and following ICaL block (red). [*C(ii)*] Individual cell response (grey), median response (black) shown. [*C(iii)*] ΔAPD in individual AP models. The black line in [*A(iii)*], [*B(iii)*] denotes the minimum ΔAPD90 possible (e.g. a 280 ms) AP which shows an HR response during 500 ms pacing must have prolonged by at least 220 ms (500–280 ms). Consequently, the equation of the black line is defined as: max ΔAPD90 = 500 − APD90. [*C(iii)*] ΔAPD90 with nifedipine vs. baseline APD90. The black line in [*C(iii)*] denotes the maximum ΔAPD90 possible (e.g. a 280 ms) AP has a maximum ΔAPD90 of −280 ms. Consequently, the equation of the black line is defined as: max ΔAPD90 = − APD90. Dashed lines in histograms indicate ΔAPD90 = 0. Statistical comparison between the paired data sets shown in [*A(ii)*], [*B(ii)*], and [*C(ii)*] was made using the Wilcoxon matched-pairs signed-rank test (****P < 0.001).

Dofetilide and nifedipine were assumed to act as single-pore IK(r) and ICa(L) blockers, respectively.^34^ To model effects of 30 nM dofetilide and 1 μM nifedipine, 30% IK(r) inhibition and 60% ICa(L) inhibition were introduced in all variants of the experimentally calibrated model population. A normally distributed error (0 ± 23 ms mean ± SD) was added to each APD_90_ and ΔAPD_90_ value to reproduce the variation in experimental measurements. *Figure 5A* shows the simulated DMSO effect indicating clustering around the zero-value similar to that shown in *Figure 3A(iii)*. Simulation of the dofetilide effect is shown in *Figure 5B*; the example APs in *Figure 5B(i)* indicate two responses: (i) APD_90_ increase and (ii) failure of repolarization similar to the experimental HR group. In some cell models, IK(r) block had minimal effects on APD [*Figure 5B(ii)*]. The relationship between the baseline APD_90_ and the dofetilide response indicates a large range of ΔAPD_90_ responses along with a sub-population of HRs [*Figure 5B(iii)*]. A subgroup of the HR models showed EADs during the plateau phase [2.6% of models, Supplementary material online, *Table S4A(ii)*].

Simulation of the nifedipine effect is shown in *Figure 5C*. The range of ΔAPD_90_ values [*Figure 5C(iii)*] showed a similar distribution to that seen experimentally [*Figure 3C(iii)*]. The average effect of the two drugs and the proportion of HRs with dofetilide (Supplementary material online, *Table S4*) was similar to the experimental data (Supplementary material online, *Table S2*) with the possible exception of EAD occurrence with dofetilide (2.6% of models vs. potentially <2% experimentally).

### 3.6 Characterization of sub-populations of AP models with different response features

**Figure 6:**
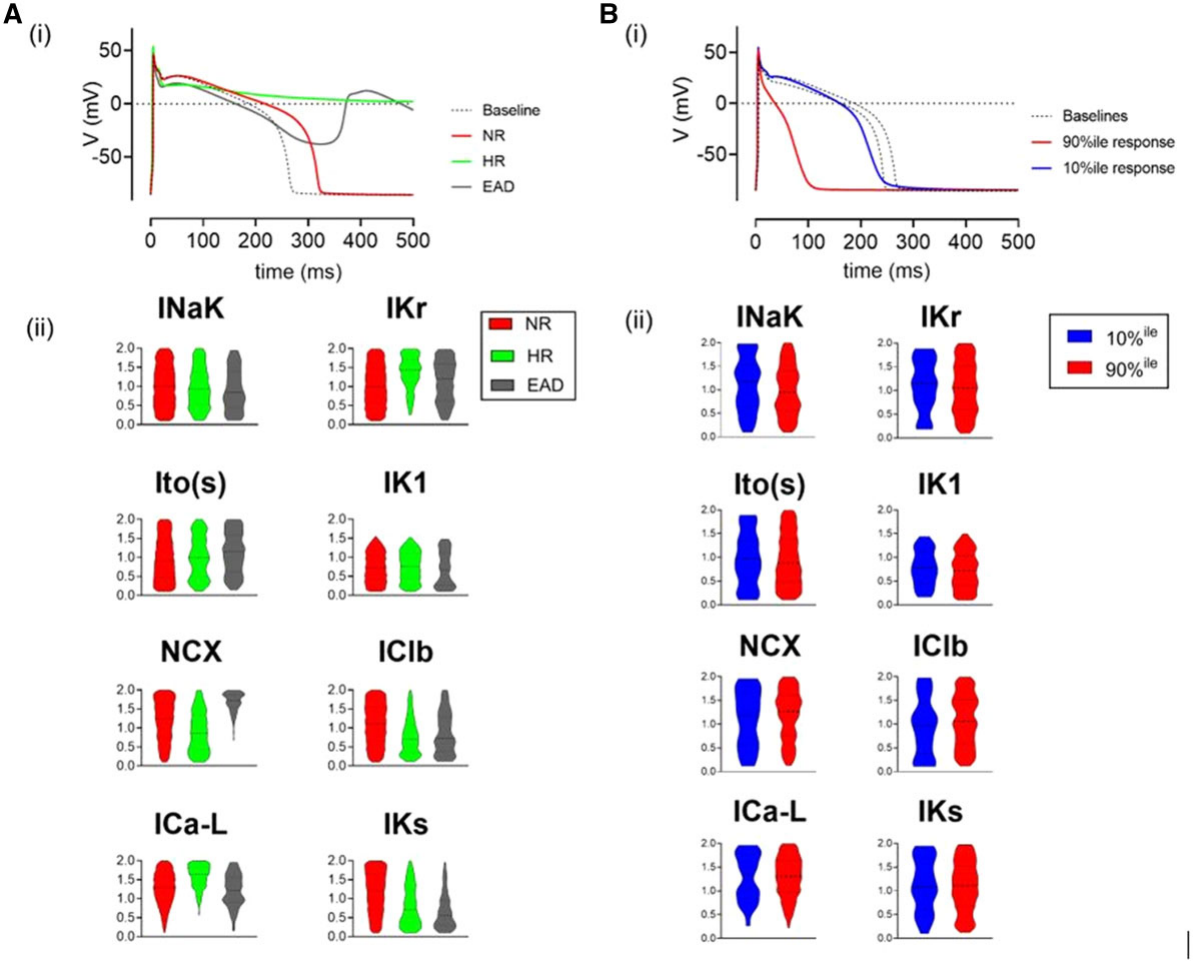
[*A(i)*], Model APs at baseline (dashed line) and typical responses to 30% IKr reduction. NR (red) shows modest AP prolongation, HR (green) shows failure of repolarization. EAD (grey) responses (defined here as positive deflection of membrane potential ≥10% of AP amplitude) are also shown. [*A(ii)*] Violin plot comparison of mean ion channel conductance for each AP subgroup. [*B(i)*] Example traces of two typical AP responses to 60% ICa(L) block. Baselines (dashed lines) and 10%^ile^ (blue) and 90%^ile^ (red) responses are shown. [*B(ii)*] Violin plot comparison of typical ion channel conductance in low (10%^ile^) and high (90%^ile^) responders to ICa(L) block.

Based on their response to IK(r) block, AP models were categorized into three subgroups: (i) NR, (ii) HR, and (iii) EAD. Representative APs and relative conductances for the three sub-groups are shown in *Figure 6A*. Comparison of the baseline APD_90_ values of NR and HR archetypes show similar separation to that seen experimentally (250 ± 46 vs. 315 ± 33 ms). Despite the similar baseline AP waveforms, the underlying conductances were different, with HRs having greater IK(r) and ICa(L) and less INCX, ICl(b), and IK(s). Interestingly, the EAD models had a similar profile to the HRs but with higher NCX. The timecourses of ICa(L), IK(r), INCX, and IK(s) for these three subgroups are shown in Supplementary material online, *Figure S5*. These indicate that a larger ICa(L) and consequently more positive plateau will delay activation of IK(r) and generate the HR and EAD waveforms. Note that absolute values of the plateau phase cannot be determined from optical signals and therefore this cannot be confirmed from the experimental data (*Figure 3A*).

Two subgroups of AP models with the largest (>90th percentile) and smallest (<10th percentile) responses to ICa(L) block are shown in *Figure 6B*. These were selected from models with baseline APD_90_ close to the median. Interestingly, these extremes were not generated by separated groups of ICa(L) conductances. As shown in more detail in Supplementary material online, *Figure S6*, the models with the largest responses to ICa(L) block had a large range of ICa(L) conductances.

In contrast, the minimal and maximal responses to IK(r) block were a consequence of very low (∼10%) and high levels (∼150%) of IK(r) conductance, respectively (Supplementary material online, *Figure S7*).

### 3.7 Electrical coupling of single-cell AP models

On the assumption that the experimentally calibrated single-cell AP models represented the biological range within the LV, the consequences for myocardial electrophysiology were investigated by creating a simplified one-dimensional cell array model by electrically coupling a random sample of 30 myocytes. On electrical coupling, the electrotonic effects narrowed APD_90_ variation across the 30 AP models changed from 212 ± 54 to 199 ± 1.5 ms (Supplementary material online, *Figure S8*). The range of Ca^2+^ transient amplitudes was also reduced (444 ± 156 vs. 385 ± 12 nM, Supplementary material online, *Figure S8*). The drug challenges were repeated in the coupled and uncoupled models (Supplementary material online, *Figure S8C*). The increase in APD on IK(r) block in the uncoupled models was larger than when they were coupled (25 vs. 19 ms). The mean decrease in APD on ICa(L) block was more comparable (−68 vs. −71 ms). Incorporating up to 80% models with HR (30% normal AP) in the coupled tissue model leads to a normal AP waveform but increases the value of APD90 as shown in Supplementary material online, *Figure S9*. Thus, it appears that abnormalities in AP repolarization are suppressed by inter-cell coupling. Furthermore, numerical simulations indicated that in contrast to the prototypical models of coupling and synchronization phenomena in biological and man-made dynamical systems,^35^ there is no critical coupling strength at which the coupled models synchronized. The range of APD90 values measured in our coupled simulations monotonically decreased as the gap junctional conductance is increased as illustrated in Supplementary material online, *Figure S11*.

## 4. Discussion

As shown previously^36^ voltage-sensitive dyes enable high-high time resolution APD measurements in isolated ventricular cells. Due to the uncalibrated nature of the fluorescence signal, absolute voltages could not be resolved, but timing of the different phases could be accurately assessed using 10 kHz acquisition rate. In this study, their use along with automation of data collection and analysis facilitated the measurement of AP characteristics from a sample of 100–200 isolated cardiomyocytes from defined regions of the LV free wall in a single session. This revealed marked variability in AP waveforms (predominantly later repolarization) in healthy LV cells stimulated at 2 Hz. This variation is not thought to reflect damage associated with cell dissociation because: (i) no relationship between cardiomyocyte yield and median/IQR APD_90_ was observed, (ii) all cells had a narrow range of AP rise times, and (iii) all cells had beat-to-beat APD values that varied <2%.^31^ The APD_90_, APD_30_, and rise times had skewed distributions and so median and IQR were used to quantify variability. The IQR of APD_90_ was 40–50 ms (median APD_90_: 250 ms); IQR decreased to 25–30 ms at 3 Hz stimulation (median APD_90_: 210 ms) and increased to 75–95 ms at 1 Hz (median APD_90_: 340 ms). This variability was considerably larger than the endo-epicardial and apical-basal differences in median APD_90_ (17 and 30 ms, respectively at 2 Hz) indicating that regional variation in APD_90_ accounts for only 5–6 ms (∼10%) of the ∼50 ms IQR. This is consistent with the similarity of the IQR value (40–50 ms) in samples from a limited area (LV basal epicardium) and the whole LV free wall. The average IQR from a limited region of a single heart (50 ± 17 ms) was comparable with the IQR of median APD_90_ from a sample of 17 hearts (45 ms), thereby demonstrating for the first time that intra-heart variation in APD_90_ may be at least as large as that seen in a population (i.e. between hearts). The spike and dome morphology of the AP differs between sub-endocardial and sub-epicardial regions,^37,38^ but the resolution of these features in optical signals from rabbit hearts is currently poor and cannot be used reliably.

In humans, although population QT_c_ intervals (and therefore the underlying average coupled APD_90_ values) are normally distributed,^1^ the single-cell APD_90_ within individual hearts is almost certainly not normally distributed. The cause of this variation in a genetically identical population of cells is unknown but may be due to inter-cell variations in expression or from different stoichiometries of alternatively spliced sub-units or co-assembly with β-subunits and therefore maximal conductance of ion channels/exchangers/pumps. Alternative causes for variation in APD include variations in the kinetics of ion channels.^9^ Differences in kinetics arise from genetically determined single nucleotide polymorphisms, so are unlikely to cause differences in ion channel activity in cells from the same individual.

To investigate the basis for the variation, the effects of ion channel inhibition were compared between cells. IK(r) inhibition by dofetilide (30 nM) caused a range of ΔAPD_90_ ranging from 0 to 50 ms (mean 30 ms). But in 10–15% of cells from each heart dofetilide caused repolarization to fail (at least over the cycle time of 500 ms). These prolonged plateau phases did not exhibit EADs. HR cells had baseline APD_90_ values comparable with the whole population consistent with the concept seen experimentally and in computational models, that similar APD waveforms can arise from quite different combinations of ion channel expression.^8,39,40^

The addition of the ICa(L) blocker nifedipine (1 µM) caused a range of ΔAPD values ranging from <10 to −170 ms with a mean value of −97 ms. The effect of nifedipine was considerably less in cells with low baseline APD values. The diversity of responses to a standard specific block and the complex relationships between baseline APD_90_ and ΔAPD_90_, in addition to a subgroup of cells with exaggerated responses to IK(r) block, indicate a wide naturally occurring range of cellular electrophysiological phenotypes even within cells with similar baseline APD_90_ values.

To test the hypothesis that variability of protein expression/activity underlies this electrophysiological heterogeneity experimentally would require measurements of ion channel expression or function linked to a baseline APD in many cells (>100) from the same heart. While this is not possible with current technology, recent studies have indicated a wide range of ion channel conductances in a limited number of isolated cells (<10) across several hearts^40,41^ and highly variable levels of single-cell ion channel mRNA measurements have been reported.^42^ This single-cell RNA sequencing methodology would need to be linked to single-cell electrophysiological assessment and scaled up considerably to address the problem at hand. The alternative, used here is to employ computational modelling to test the impact of variation in ion channel/pump/exchanger activity in an analogous approach to that used to explain inter-individual variability.^7,8,39^

### 4.1 Computational modelling of variability

A population of models was created by random variation in 8 key conductances associated with the rabbit ventricular AP using techniques similar to previous studies.^6,8^ An initial wide limit of variation (10–200%) was used to generate models with APD_90_ values that overlapped with those obtained experimentally. A subset of models that generated APs was selected to match the median and distribution of APD_90_ values observed experimentally. The use of APD_90_ as the initial repolarization biomarker for calibration of the model was supported by the resultant correspondence of the other AP parameters including an index of AP shape (triangulation). The %IQR coefficient of the APD_90_ at 2 Hz for the 4471 experimentally calibrated models was 24%, close to the average reported experimentally (19–25%). Strikingly, this variability of APD_90_ was achieved with %IQR coefficients ranging from ∼99% [Ito(s)] through 80–90% [IK1, IK(s), IK(r), ICl(b), INaK] to 75% (INCX), and 50% [ICa(L)]. Values for inter-cell variation of ion channel expression within a single heart have not been documented, but an electrophysiological study of ICa(L) in ventricular cardiomyocytes across different individuals and species reported a %coefficient of variation (100× SD/mean) of ∼55%,^41^ this approximates to an %IQR coefficient of 45%, i.e. similar to the ∼50% reported for ICa(L) in our model population. Further support for the existence of highly variable inter-cell conductance values comes from AP clamp studies indicating a 6× range of values of IK(s) in cells with similar APD_90_ values, albeit across different hearts.^43^ In summary, where investigated, inter-cell variation in ionic conductance appears large and suggests that precise control over individual ion channels/exchanger is not a prerequisite for stable APs. Instead, as this study indicates, the relative expression of conductances [ICa(L), INCX, IK(r), IK(s) INaK, and ICl(b)] is crucial for viable single-cell APs. Experimental corroboration of these interrelationships is needed. The largest scope for variation was for Ito(s), a channel considered important for repolarization reserve.^44^ This result does not imply that this variation in Ito(s) exists in single cells, but that the AP biomarker used (APD_90_) is not sensitive to large variations in Ito(s). Interestingly, despite this variation, Ito(s) conductance was positively correlated with ICa(L) in the models (Supplementary material online, *Figure S9*). The experimentally calibrated models reproduced the range of AP waveforms in terms of the timing of different AP phases. Absolute levels of diastolic, peak, and plateau voltages are additional important repolarization biomarkers but are not available from optical measurements. Nevertheless, the refinement of the models based on the AP waveform alone resulted in a narrow range of diastolic and peak potentials (diastolic *E*_m_ −85.5 ± 1.1 mV, peak Em 49.0 ± 4.2 mV) which overlap with reports from isolated rabbit ventricular myocytes.^18,19,31^ The consequences for E-C coupling can be inferred from the predicted model Ca^2+^ transient (Supplementary material online, *Figure S8*).^18^ This showed a large variation of amplitude which would be expected to result in a large range of contraction. How this is reconciled with the behaviour in the intact myocardium is discussed later. The predicted mean value for Ca^2+^ transient amplitude (∼400 nM) is similar to previously published experimental values at 37°C, 2 Hz stimulation.^19,45^ It was noted that the inter-cell variation in Ca^2+^ transient amplitude was large in some instances peak systolic Ca^2+^ could reach ∼1.5 µM (Supplementary material online, *Figure S8*), a value well within the range reported in previous publications.^19,45^ It will be of interest to adopt multivariate regression methods that have been used for local sensitivity analysis^26,27,30^ to also solve the problem of parameter value estimation. This latter issue is the main concern of the current computational analysis. Regression analysis has already been successfully applied to synthetic data for constraining parameters in electrophysiological models of cardiac cells by Sobie and Sarkar.^46^ However, the latter method cannot be applied directly to the current experimental data since the number of conductances to estimated is greater that the number of linearly independent biomarkers that can be measured from the dataset.

### 4.2 Co-regulation of ionic conductances

The largest positive correlation in the model conductances was between ICa(L) and IK(r) (all correlations summarized in Supplementary material online, *Figure S10*), indicating that viable APs required a balanced expression of at least these two ion channels. A strong correlation in mRNA signals for ICa(L) and IK(r) was reported in a human population and induced pluripotent stem cell (iPSC)-derived cardiomyocyte cell lines^7^ despite large variations in individual mRNA signals. Ballouz *et al.* used modelling to show that linking ICa(L) and IK(r) conductance limits the range of APDs despite large ranges of absolute values. Furthermore, this linkage reduced the incidence of EADs in response to IK(r) block in the O’Hara *et al*.^47^ model of the human ventricular AP, an observation consistent with the very low incidence of EADs in this study.

A previously unreported positive correlation was observed between ICa(L) and the baseline chloride conductance. The participation of chloride channels in cardiac electrophysiology is well documented,^48,49^ including their contribution to outward (repolarizing) current, although the molecular identity of the dominant channel in the ventricle is unclear.^49^ The negative correlation of ICa(L) and INCX observed in the model dataset has not been previously reported, the mechanistic basis for this is uncertain, but may be linked to the maintenance of the plateau phase to which both ICa(L) and INCX contribute. Higher INCX conductances in conjunction with a higher ICa(L) in small group (2%) of AP models demonstrating EADs (*Figure 6*) suggests that a reciprocal ICa(L)-INCX relationship may help reduce the incidence of EADs. There is an emerging literature concerned with coordinated expression of ion channels/exchangers/pumps in cardiac cells,^7,40,42,50^ and a comparable role for specific conductance ratios in constraining neuronal electrical activity.^10,12,51,52^ A candidate mechanism for co-regulation of genes in neurons and cardiac muscle is intracellular Ca^2+^ which can control specific transcription factors and co-regulators via nuclear and cytoplasmic Ca^2+^ binding proteins.^42^ A direct link to Ca^2+^ channels has been provided by studies showing that a C-terminal fragment of the L-type Ca^2+^ channel acts as a transcription regulator for a large group of genes including repolarising potassium channels in neurons.^53^ Interestingly, in addition to up-regulation, the C-terminal sub-fragment-mediated down-regulation of a group of genes including SCL8A1, a gene encoding a Na/Ca exchanger, paralleling the inverse correlation between ICa(L) and INCX observed in this study. The inverse relationship between ICa(L) and NCX activity initially appears counter-intuitive given their roles in Ca^2+^ homeostasis, but stoichiometric regulation of NCX by intracellular Ca^2+^ is a powerful regulator of NCX activity^54^ and therefore key to the interplay between Ca^2+^ influx and efflux on a beat-to-beat basis. These and other studies have given rise to the concept of a ‘microtranslatome’^42^ to describe a mechanism of co-translation/regulation of a group of genes that regulate the relative levels of a specific group of proteins/conductances in a cell to generate a functional E-C coupling process.

### 4.3 Correspondence between the 4471 AP models and experimental responses to ion channel block

The level of channel inhibition used to model dofetilide effect on IKr (30%) and nifedipine effect on ICa(L) (60%) was estimated by approximating the median change in APD observed experimentally (Supplementary material online, *Table S2*) with that calculated from the 4471 models (Supplementary material online, *Table S4*). As indicated in *Figure 5*, the resultant spread of data and the relative occurrence of HR matched the experimental data well. A kernel density plot (Supplementary material online, *Figure S4*) indicates that the populations of models with distinct responses possessed distinct combinations of ICa(L), IK(r), and INCX conductances. Supplementary material online, *Figure S5*, shows that under baseline conditions the archetypal HR model had a larger Ca^2+^ transient amplitude consistent with the larger ICa(L) and lower INCX conductances. In addition, ICl(b) was lower than the NR group, thus on IK(r) inhibition, this group of AP models would have less repolarising current available at the plateau phase which may explain the HR response. The only apparent disparity from the range of experimental responses was the small but significant subgroup of AP models with EADs after IK(r) block. At 2.6%, this response was 3–4× less common than HR response in the model population, at 8.2%. Experimentally, EAD events were not observed under these conditions suggesting an incidence of <2% (Supplementary material online, *Table S2*). The EAD model subgroup had similar characteristics to the HR group except for greater INCX. As shown in Supplementary material online, *Figure S5*, the EAD archetype had the largest Ca^2+^ transient amplitude of the three subgroups under baseline conditions. Resolving the incidence of EADs experimentally would require an increase in sample size to >500 cells/heart region, or a change in experimental conditions to make the probability of EADs higher.

The experimentally calibrated set of models reproduced the range of electrophysiological responses to ICa(L) block seen experimentally. In 50% of models with an APD_90_ within the IQR, ICal(L) block caused a large range of ΔAPD. This group had APD_90_ values ranging from 200 to 300 ms supported by a large range of ICa(L) values balanced by IK(r) and would support a similarly large range of Ca^2+^ transient amplitude and contraction. The other feature is the uniformly negligible response to 60% ICa(L) block observed in cells with naturally very short APD_90_ (<200 ms, see *Figure 3*), the *in silico* model indicates that this behaviour is replicated by AP models with much lower ICa(L), a conclusion that supports the observed large range of ICa(L) expression in ventricular cells.^41^

### 4.4 Behaviour of the AP models when coupled as a functional syncytium

Electrically coupling a group of 30 randomly selected AP models caused APDs to become shorter and more uniform. This phenomenon has been previously reported in electrically coupled cell pairs and was explained by asymmetry of current required to increase a shorter APD compared to that required to decrease a longer APD.^55^ A pair of isolated cells with different Ca^2+^ transient amplitudes also had these differences minimized by electrical coupling,^55^ again in agreement with the reduction of Ca^2+^ transient amplitude differences after coupling individual cells reported in Supplementary material online, *Figure S8*. Thus, the electrical heterogeneity of the isolated cells reported in this study is not inconsistent with the uniform behaviour of both electrical and contractile events in the intact myocardium. Electrical coupling also reduced the magnitude of the response to IK(r) block, but interestingly, the response to ICa(L) block was unchanged. The sensitivity of the APD to the percentage of HR cells within a population of coupled cells is shown in Supplementary material online, *Figure S9*, this indicates that the system would tolerate up to 80% of cells in the HR sub-group while retaining a normal (but longer) APD when coupled. The other relationship of interest is the effect of the coupling strength on the range of APD90 values within a population of coupled cells, as shown in Supplementary material online, *Figure S11* the range of APD90 values decreased smoothly with no obvious threshold when coupling strength was increased by a factor of 100.

### 4.5 Summary

This study quantifies for the first time significant inter-cell variability in ventricular APD and provides evidence from computational modelling to show that the underlying basis is an even greater inter-cell variation in ion channel/exchanger/pump expression. The modelling supports previous studies, including those in other excitable cells, indicating that redundancy and co-relationships between key ionic conductances allow similar AP waveforms to be generated despite large inter-cell variations in individual maximal conductances.^8,10,12,39^ This degree of inter-cell heterogeneity provides a mechanistic basis for the wide range of responses to drug-induced ion channel block seen experimentally.

The level of inter-cell heterogeneity has implications for studies using single adult cardiac cells or other isolated cell preparations (e.g. iPSC-derived cardiomyocytes). Depending on the study design, the large inter-cell variation in E-C coupling means that manoeuvres to quantify changes in electrophysiology or other aspects of E-C coupling using single isolated cells will inevitably result in highly variable data (coefficient of variation, CoV ∼25%), this decreases to a CoV of 1–2% in coupled systems like trabeculae/tissue strips. Therefore, the sample size (i.e. number of hearts) required to resolve changes in electrophysiology/E-C coupling using single-cell preparations is at least 2× that required using intact tissue preparations, with some effects, e.g. HR response to ion channel block, being observed in isolated cells and not in intact tissue.

A biological rationale for naturally high cellular variability has not been established; one option is that this represents the minimum system specification to generate a functional syncytium using the engineering principle of the Good Enough System design,^56^ precise regulation of protein expression levels is not necessary and therefore energetically wasteful. Minimally, stable electrophysiology requires: (i) an AP waveform in coupled cells that generates an intracellular Ca^2+^ transient to support E-C coupling and (ii) the AP waveform should be stable across a range of heart rates, autonomic and environmental influences. Evidence for the ‘Ca^2+^-centred’ design specification was provided by a recent study on mouse heart^50^; this study indicates that this specification applies to coupled cells in the myocardium and not to single cells isolated from the syncytium. The second potential design specification of electrophysiological resilience to the altered function of one or more ion channel/exchanger/pumps through genetic variation or environmental factors^7^ provides an alternative rationale to that of energetic economy for the large degree of inter-cell heterogeneity, as shown previously, heterogeneity of Ca^2+^ signalling in the SAN is key to normal function.^57^ Further work is required to establish the key drivers that control intra-heart cellular specifications for E-C coupling.

Therefore, as previously discussed for AP waveforms in neurons^58^ and cardiac cells,^59,60^ a single model of the electrophysiology of a cell based on average values of maximal conductance is unrealistic as it ignores the complex interrelationship across the population of cells. A good example of this phenomenon is the failure of a single model with fixed parameter values to predict the range of responses to IK(r) and ICa(L) block observed experimentally. Instead, a population of models such as that developed in this study based on experimental data is necessary to explain the range of responses of single cells and ultimately the myocardium.

The translational implications of inter-cell heterogeneity in the context of expression networks are potentially far-reaching. For example, the inter-relationships indicated in this study suggest that countering the acute pro-arrhythmic effects of drug-induced inhibition of IK(r) could be achieved by inhibition of ICa(L) or INCX or augmentation of ICl(b) (or a combination of these). Congenital long QT syndrome caused by loss-of-function mutations in hERG (LQT2) should be interpreted in terms of an altered status of the expression networks rather than the effect on IKr alone. This would enable identification of cellular phenotypes prone to marked AP prolongation, which may be linked to individual susceptibility to Torsades de Pointes. Finally, in the absence of knowledge of inter-cell heterogeneity and expression networks, the changes in average protein expression or average conductance of multiple ion currents/exchangers/pumps for conditions such as the different forms of heart failure provides poor insight into the underlying disruption of expression networks and associated co-regulated proteins. Understanding the general and individual response to these environmental changes in terms of cellular heterogeneity will significantly aid the treatment design in terms of both E-C coupling and electrophysiological resilience.

## Supplementary Material

cvab375_Supplementary_Data

## Data Availability

The data underlying this article are available in the article and in its supplementary material online. Translational perspective We show that single myocytes from the same region of a heart have highly variable electrophysiology and different responses to ion-channel blocking drugs because of even higher inter-cell variation in ion-channel activity. Our data demonstrate that it is the relative activity of a single ion channel compared to others that determines electrophysiological stability in the heart. Therefore, predisposition to arrhythmias from genetic or environmental causes is not due to up- or downregulation of single ion-channels, but instead disrupted patterns of co-expression. An alternative treatment strategy therefore is to manipulate other ion-channels in the network to restore a stable co-expression pattern.
