# Supplementary material for: Electrophysiological heterogeneity in large populations of rabbit ventricular cardiomyocytes
Source: Cardiovasc Res. 2022 Jan 10;118(15):3112–25. doi: 10.1093/cvr/cvab375 (PMC9732512; doi:10.1093/cvr/cvab375)
Supplement: cvab375_Supplementary_Data [file cvab375_supplementary_data.docx]

**Lachaud et al:**

“**Electrophysiological heterogeneity in large populations of rabbit ventricular cardiomyocytes”**

# Supplementary Data

## Table S1


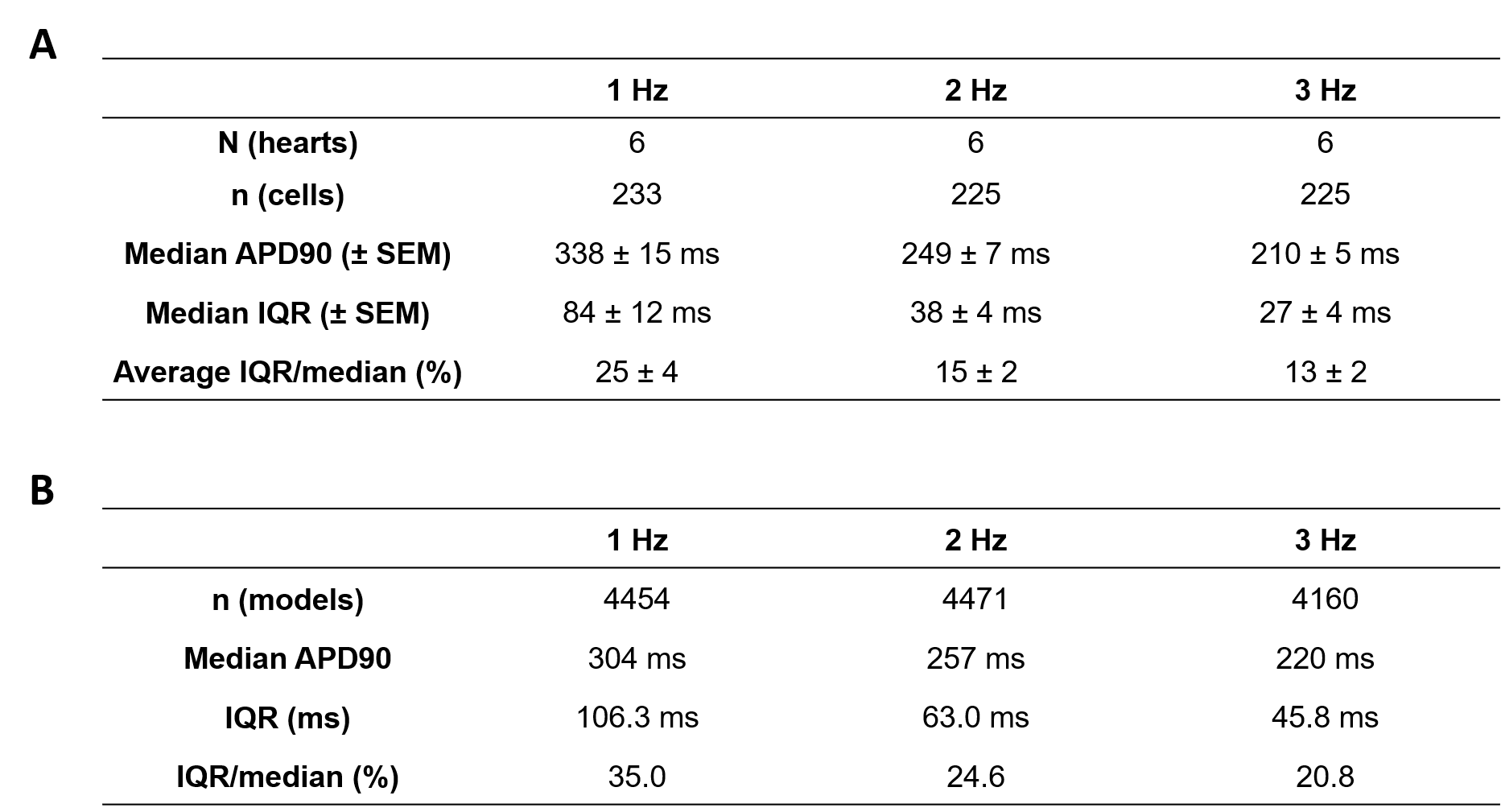


A. Median and IQR of APD90 at three pacing frequencies (1,2 and 3Hz) sampled experimentally in paired cells. B. Median and IQR of median APD90 in experimentally-calibrated models.

## Table S2


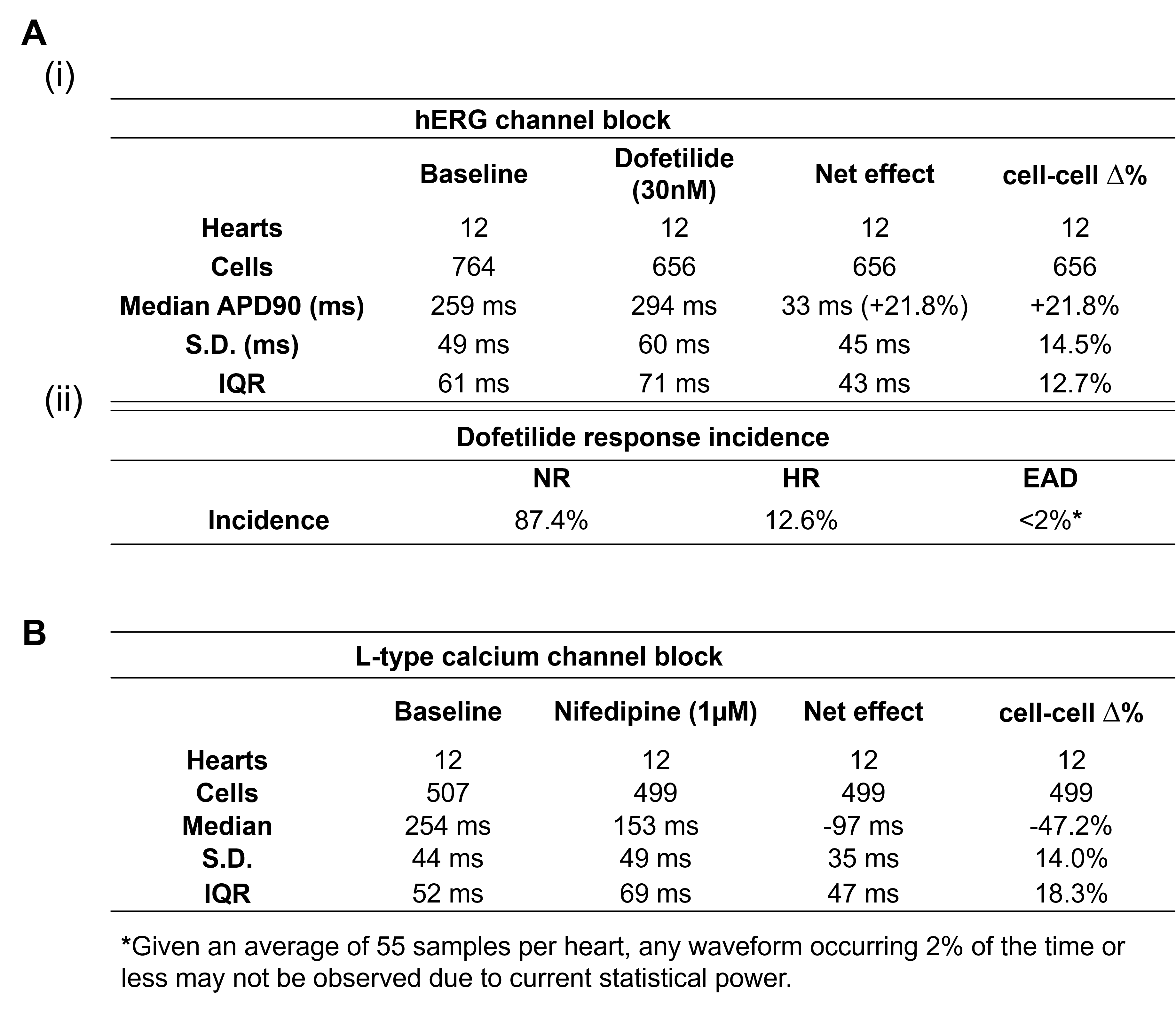


A(i) Effect of 30 nM dofetilide on APD90 in populations of epicardial cells. (ii) Percentage incidence of distinct cell responses to 30 nM Dofetilide. (NR = normal response; HR = hyper-response; EAD = early-afterdepolarisation). B Effect of 1 µM nifedipine on APD90 in populations of epicardial cells.

## Table S3


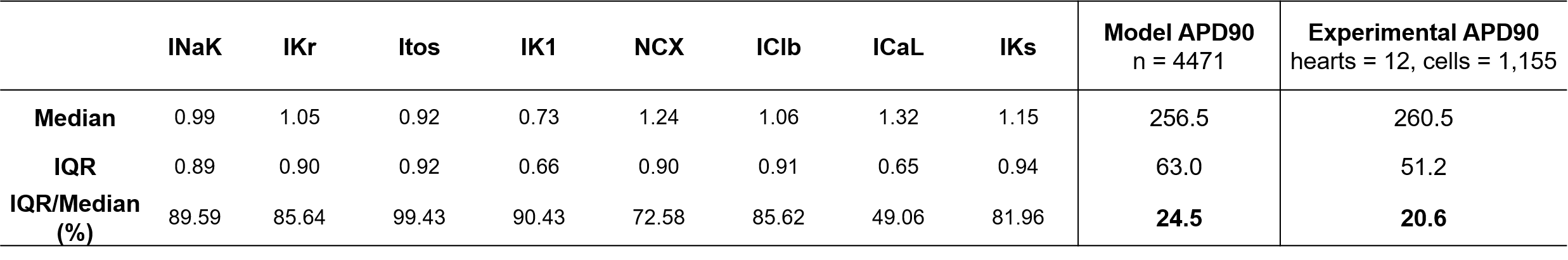


## Table S4


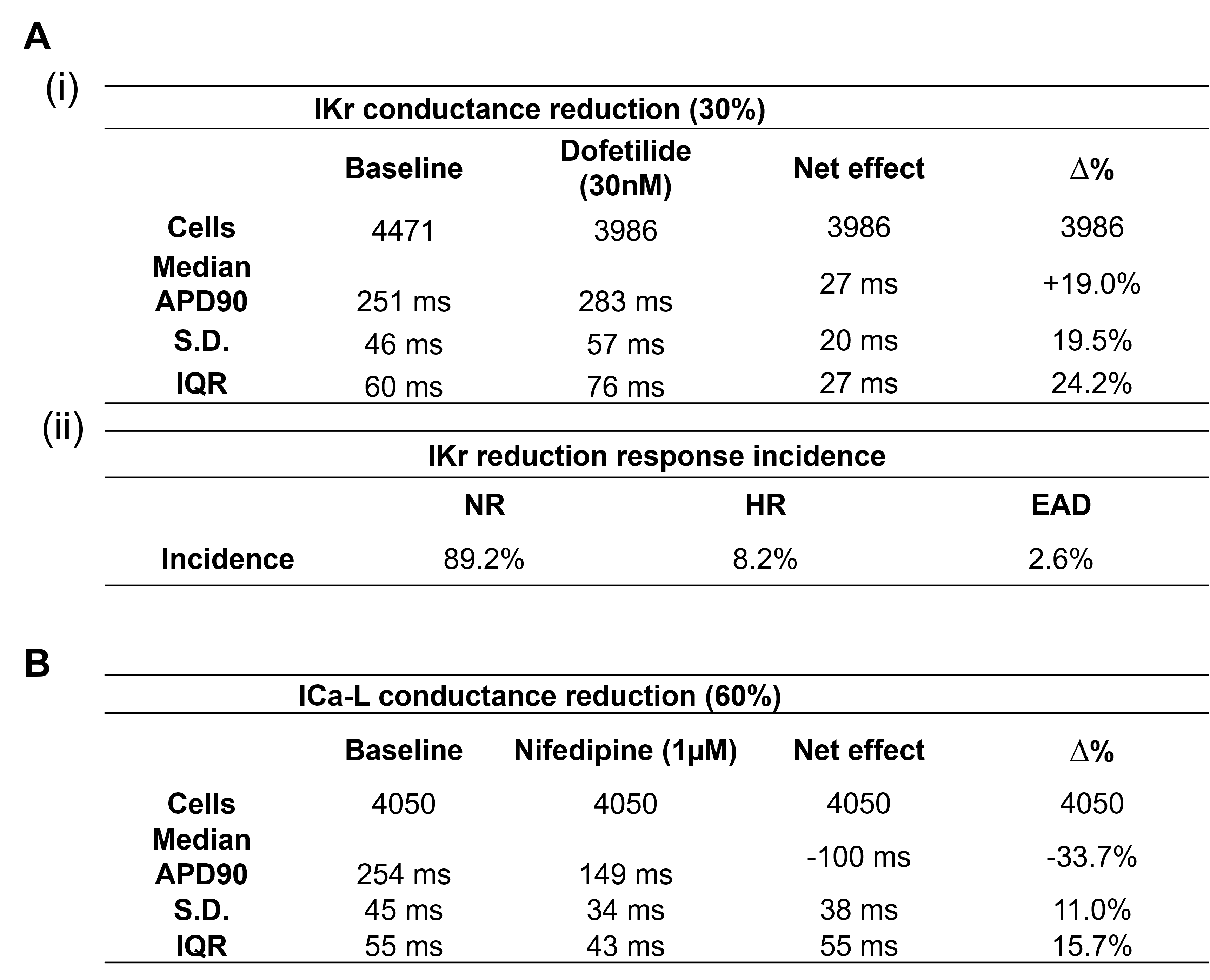


***Table S5***


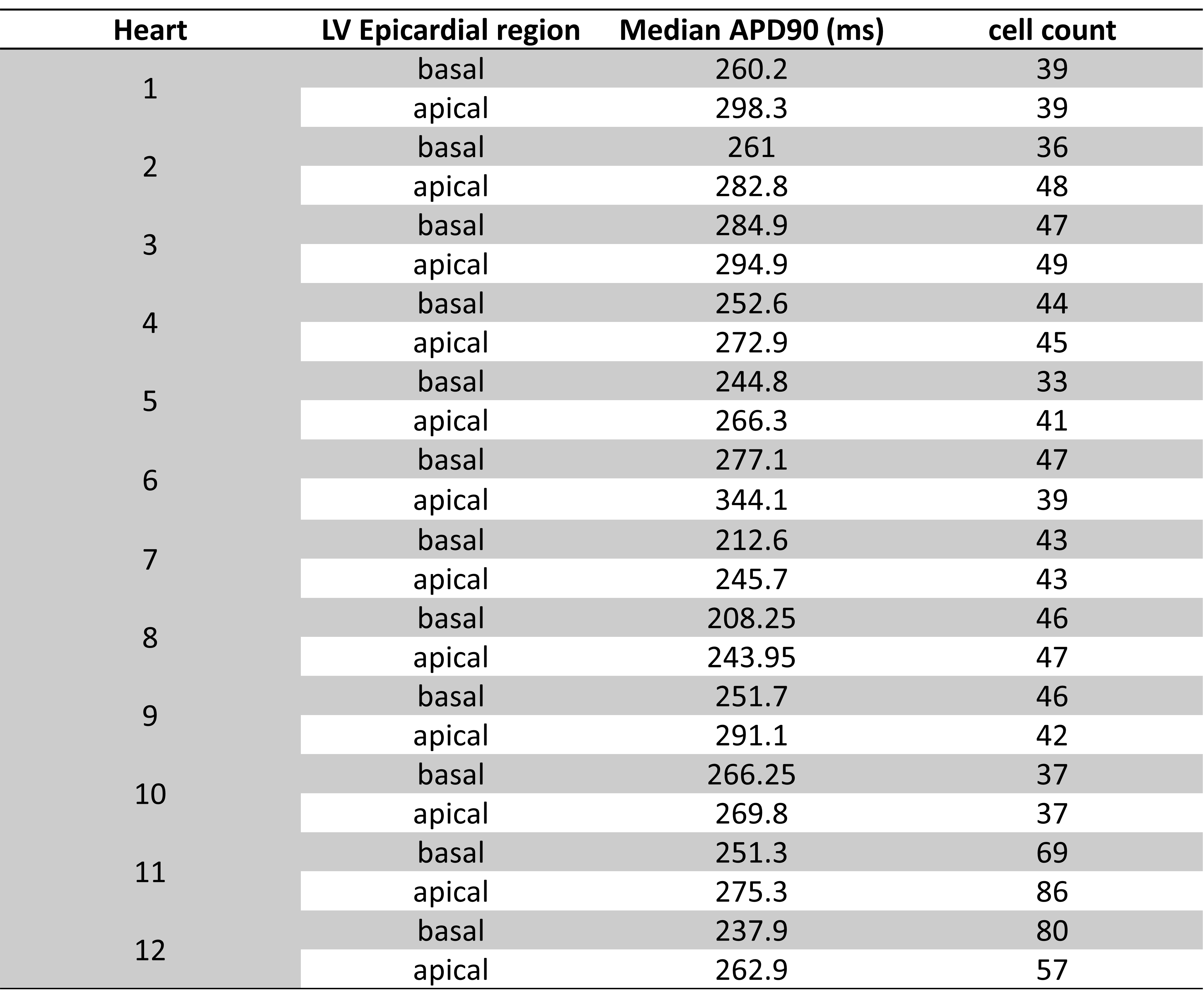


***Table S6***

***
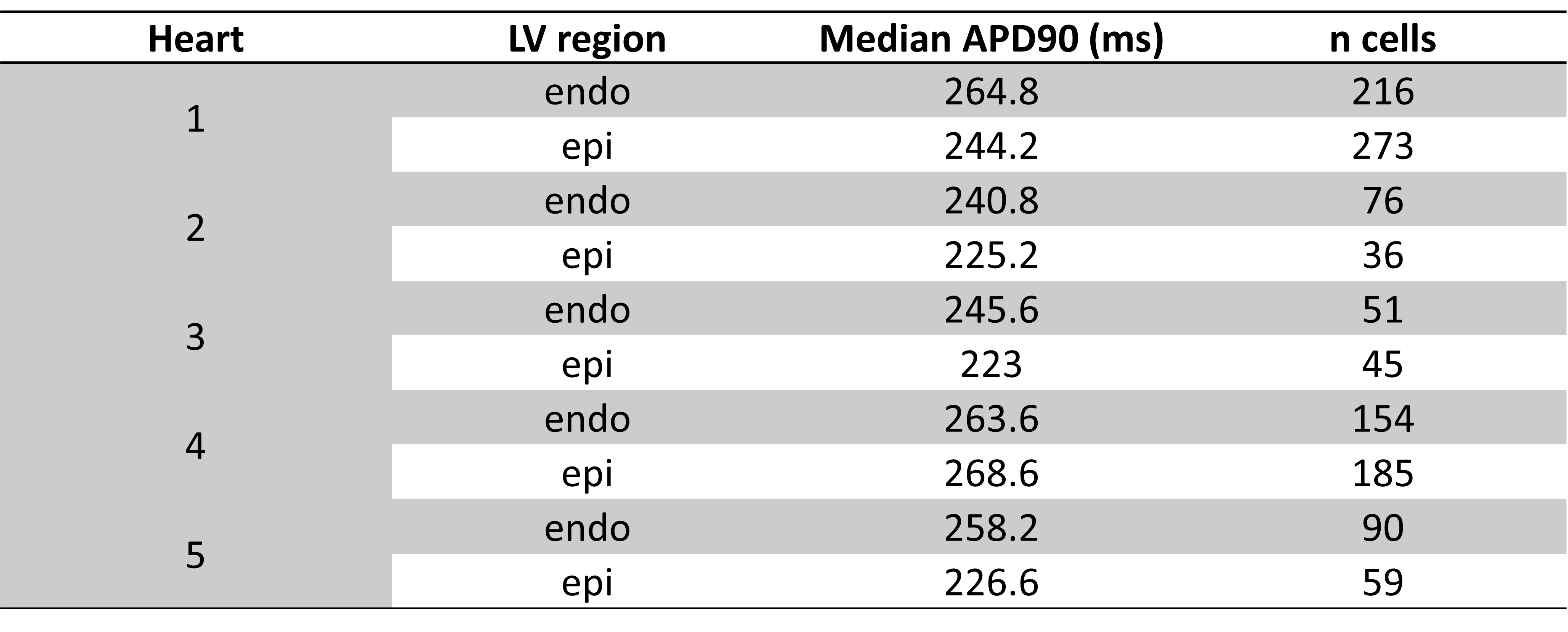
***

## Figure S1


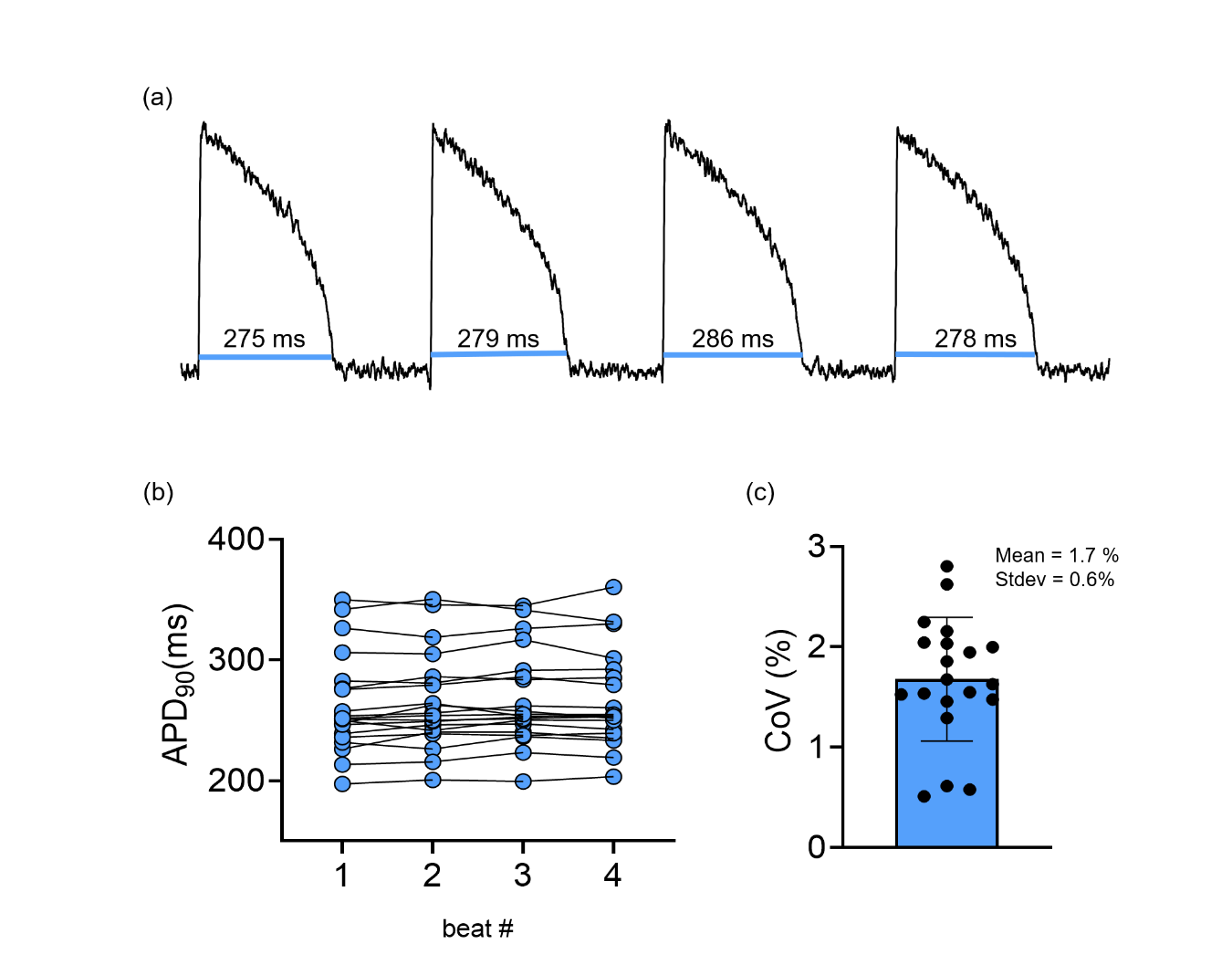


**Figure S1.** Beat-to-beat variability. (a) Action potential train in an isolated cardiomyocyte, annotated with APD90 of each transient. (b) Plot of APD90 of four consecutive action potentials in 20 cells paced at 2Hz. (c) Coefficient of variation (100×SD/mean) of APD90 measured in 4 beats.

## Figure S2


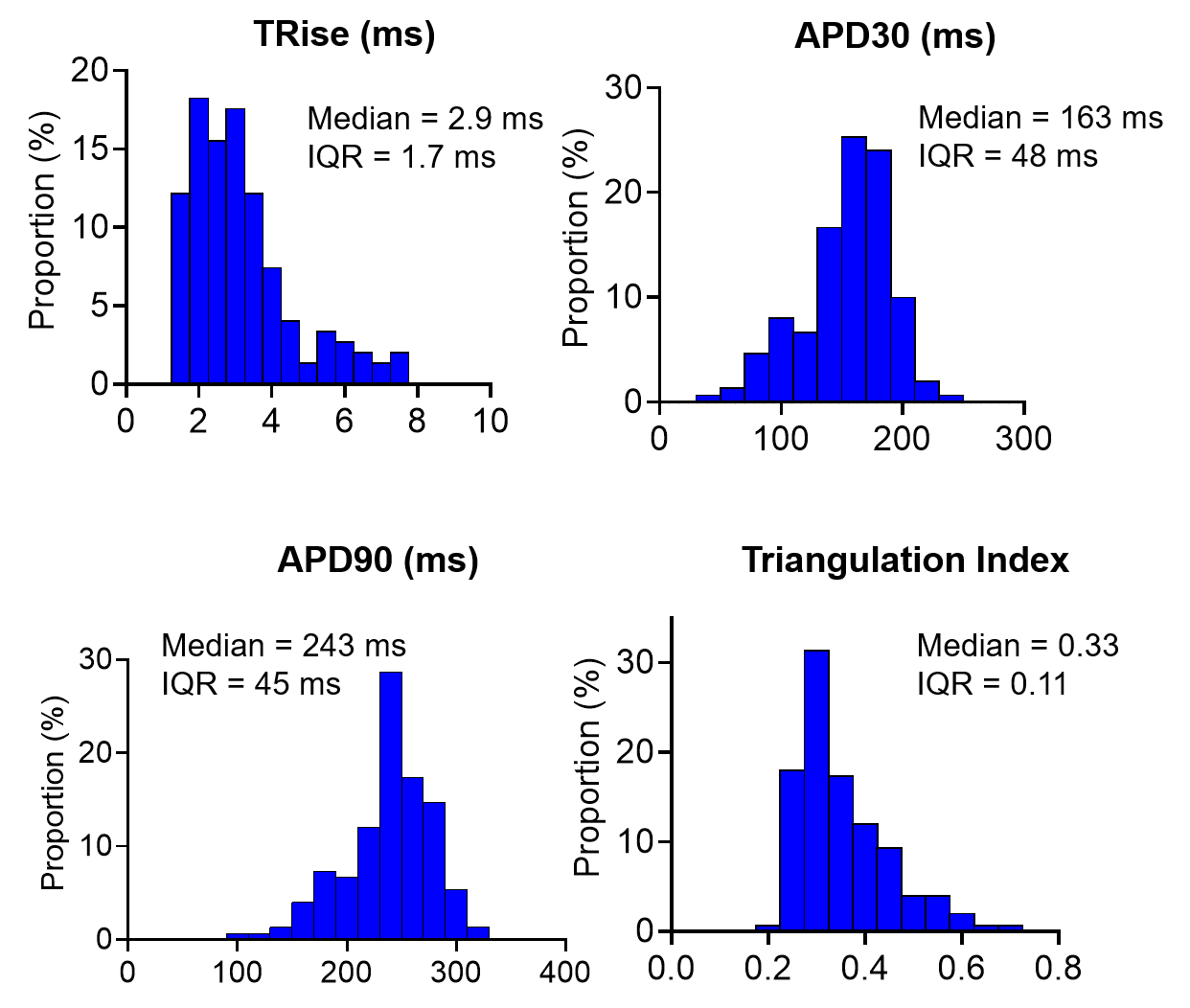


**Figure S2.** Distribution of four AP parameters measured in 150 cells originating from a single rabbit LV free wall. Triangulation index is defined as the ratio of duration of the mid- to late-plateau phase. Triangulation index = (APD90-APD30)/APD90.

**Figure S3**


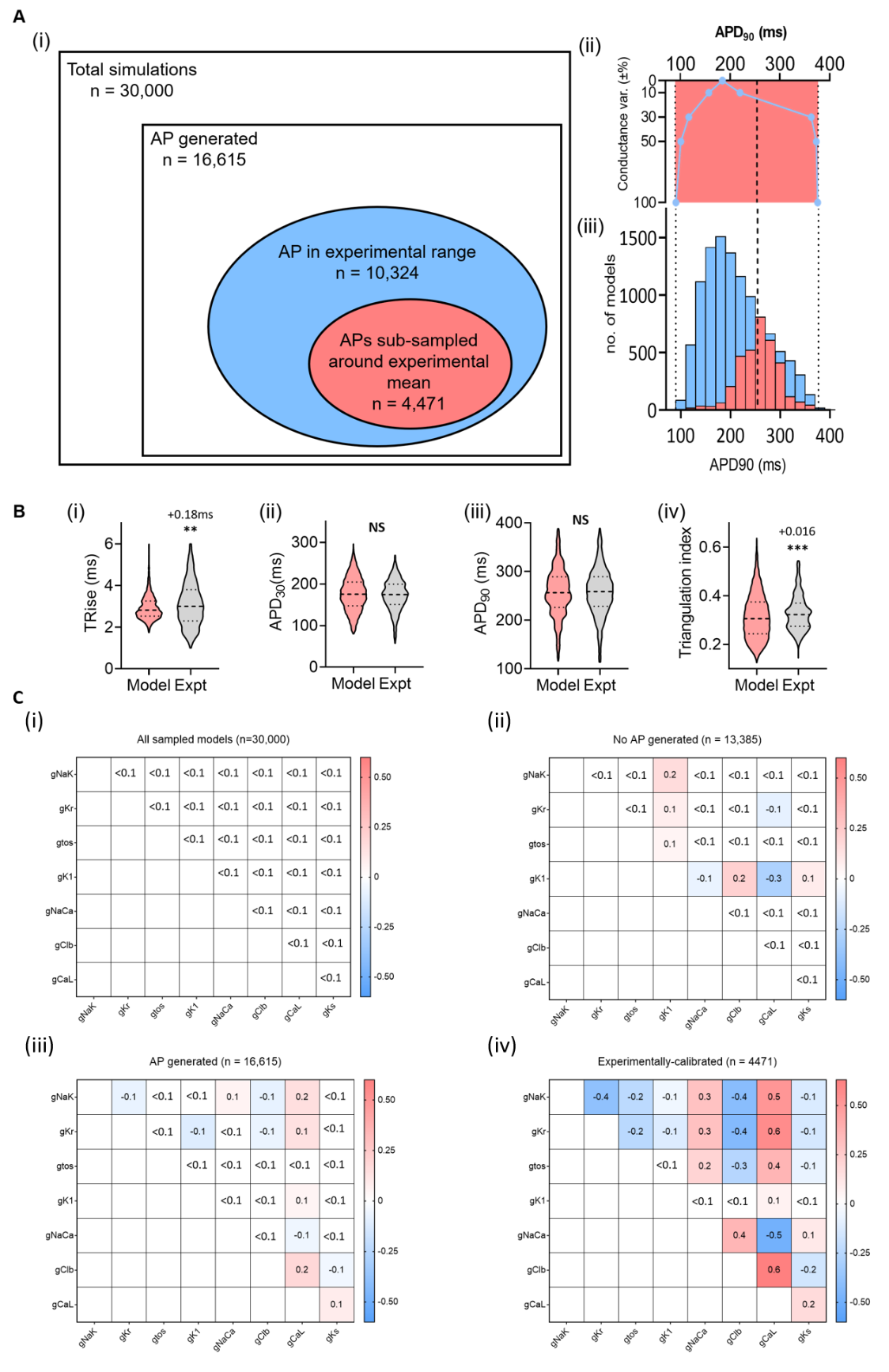


**Figure S3.** A (i) Diagrammatic representation of Shannon-derived AP population calibration process. (ii) Ranges of modelled APD90 values generated with four-step increases in conductance variation. (iii) Experimentally-calibrated Shannon model action potentials (red) selected from non-calibrated APs in experimental range (blue). B. Violin plot comparison of four AP biomarkers between model and experimental waveforms. (i) TRise was modestly longer in models (+0.2ms), APD30 (ii) and APD90 (iii), were not significantly different between model and experimental measurements. (iv) Triangulation index was significantly larger in model population, with a reported increase of 0.016. C. Partial correlation coefficient matrices of all channel conductance pairs. Coefficient values smaller than |0.1| are not shown. Non-significant (P>0.05) coefficients are also not shown. (i) All models tested. (ii) Models which did not generate APs. (iii) Models generating APs within the experimental APD90 range (min-max). (iv) Models generating APs matching the experimental APD90 distribution. Statistical comparison between the unpaired data sets shown in panel B was made using a Student’s T test (****, P<0.001).

**Figure S4**


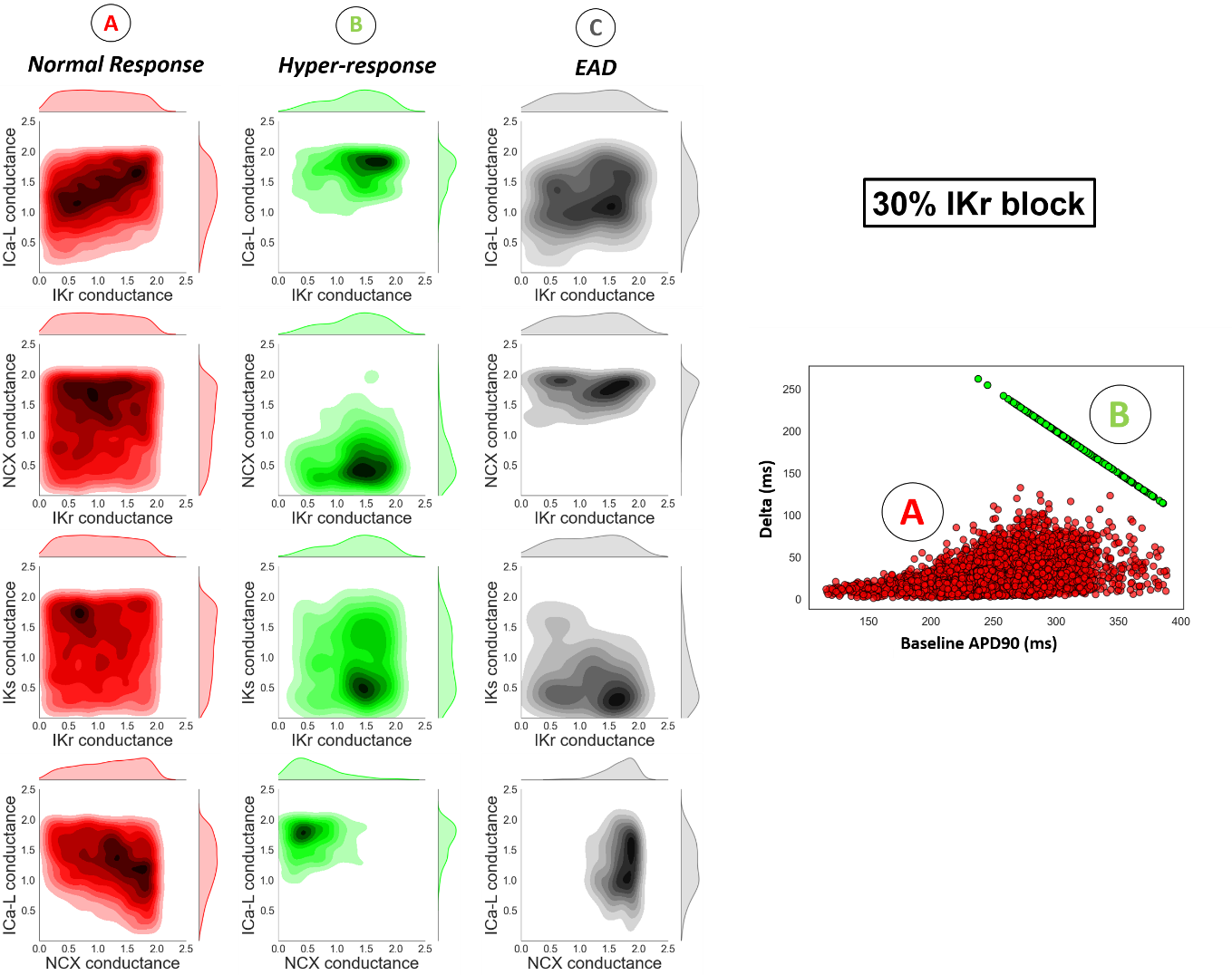


**Figure S4.** Kernel density estimation plots showing the relationship between selected ion channel pairs in each cell response type to 30% IKr block reported. (A) Normal response (APD90 < 500ms). (B) Hyper-response (APD90 > 500ms). (C) Early-afterdepolarisation (positive deflection ≥10% of AP amplitude).

## Figure S5


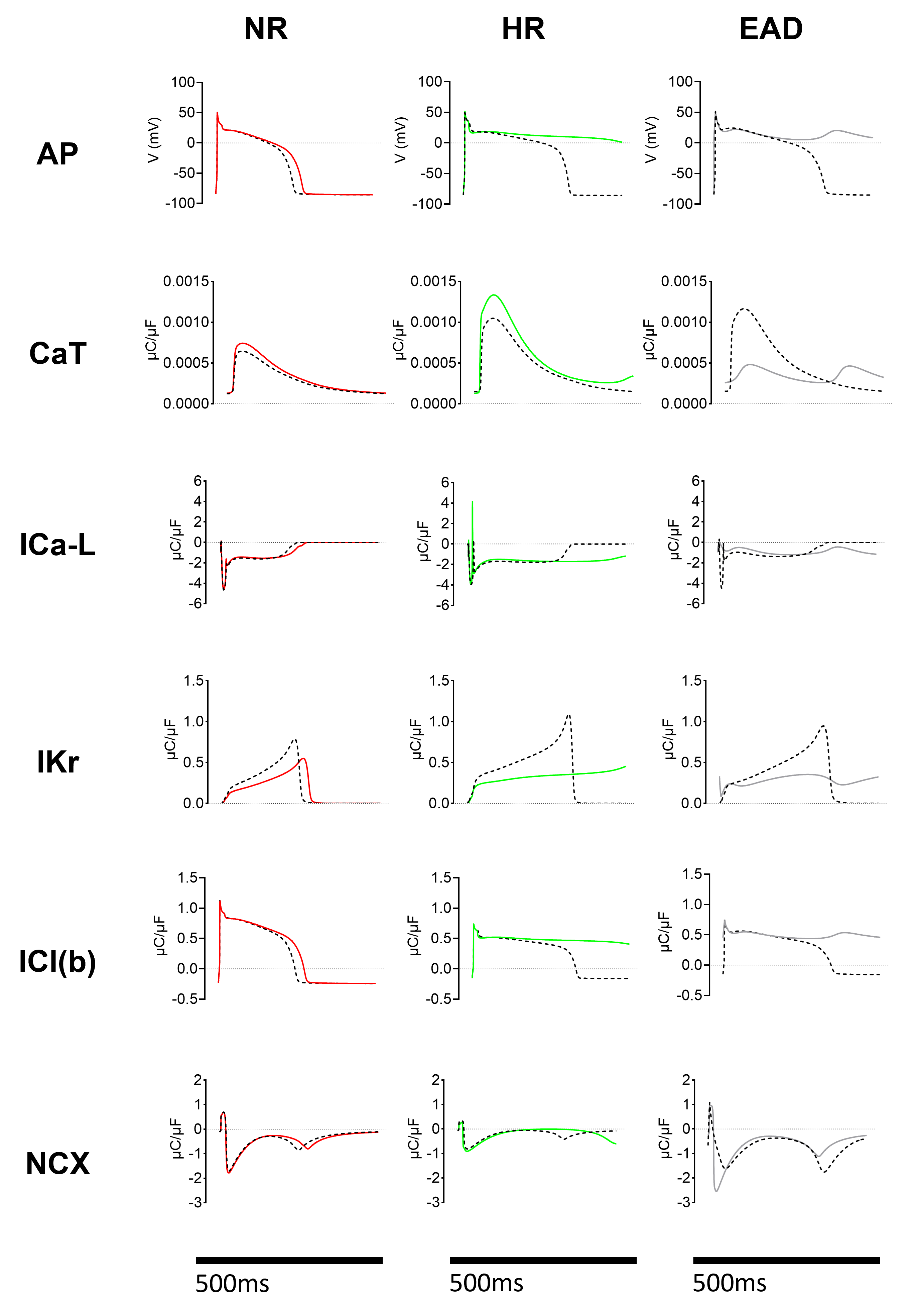


**Figure S5.** Cellular response archetypes generated from median channel conductance profiles determined in Figure 6A(i). (NR), median response to 30% IKr reduction;(HR), hyper-response (APD > 500ms) and (EAD), EAD response (plateau deflection >10% of AP amplitude). Baseline traces (dotted lines) and those with model drug effect (continuous line) are shown for each response archetype. (i) Action potential (AP). (ii) Calcium transient (CaT). (iii) L-Type calcium channel (ICa-(L)). (iv) Rapid delayed rectifier (IKr). (v) Chloride channel (ICl(b)). (vi) Sodium-calcium exchanger (NCX).

## Figure S6


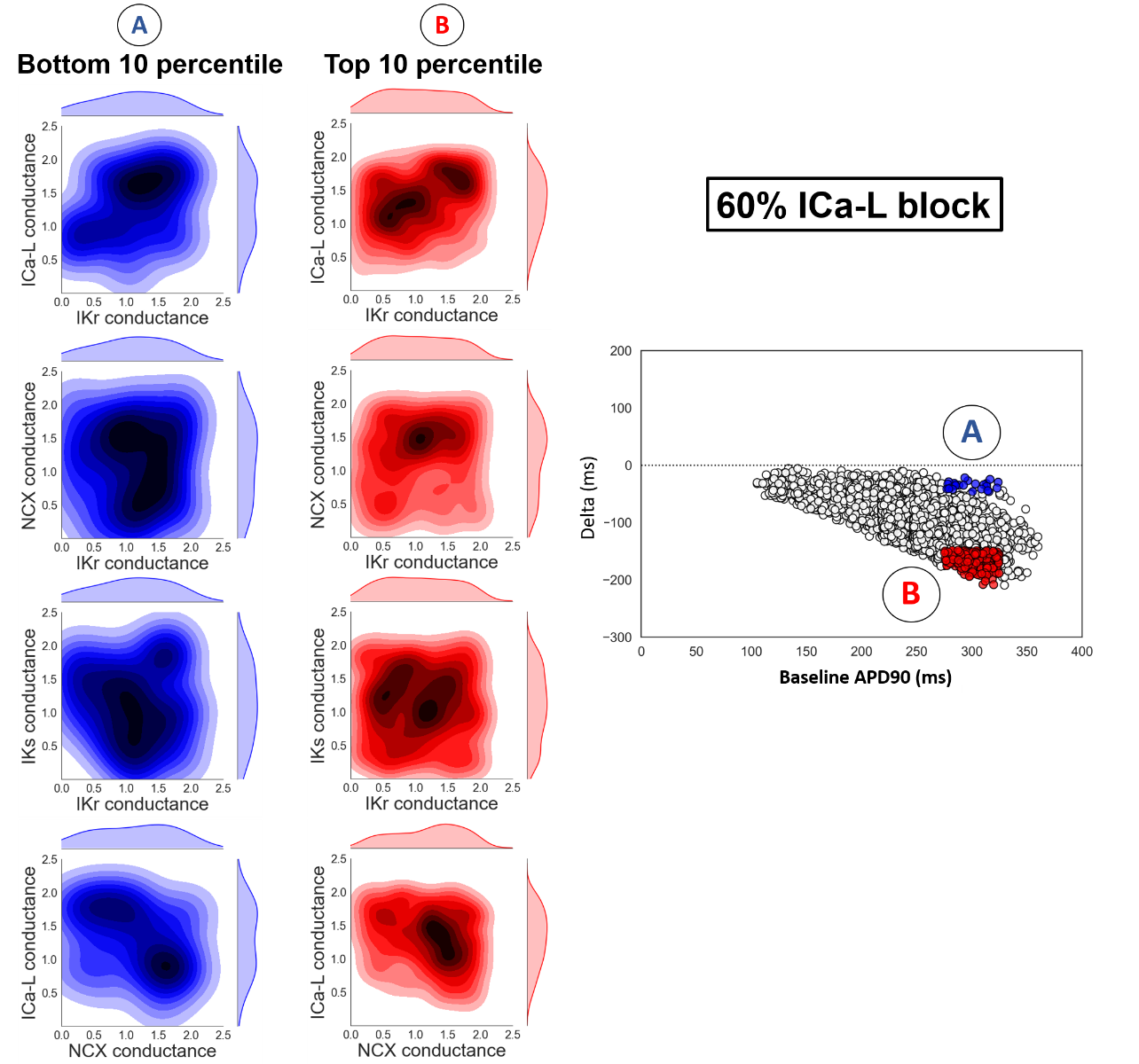


**Figure S6.** Kernel density estimation plots showing the relationship between selected ion channel pairs in two distinct responses to 60% ICa(L) reduction. (A) low (<10%-ile) and (B) high (>90%-ile) responders to ICa(L) block. Action potentials in a narrow range (285-325ms) were selected at baseline to minimise APD-bias.

## Figure S7


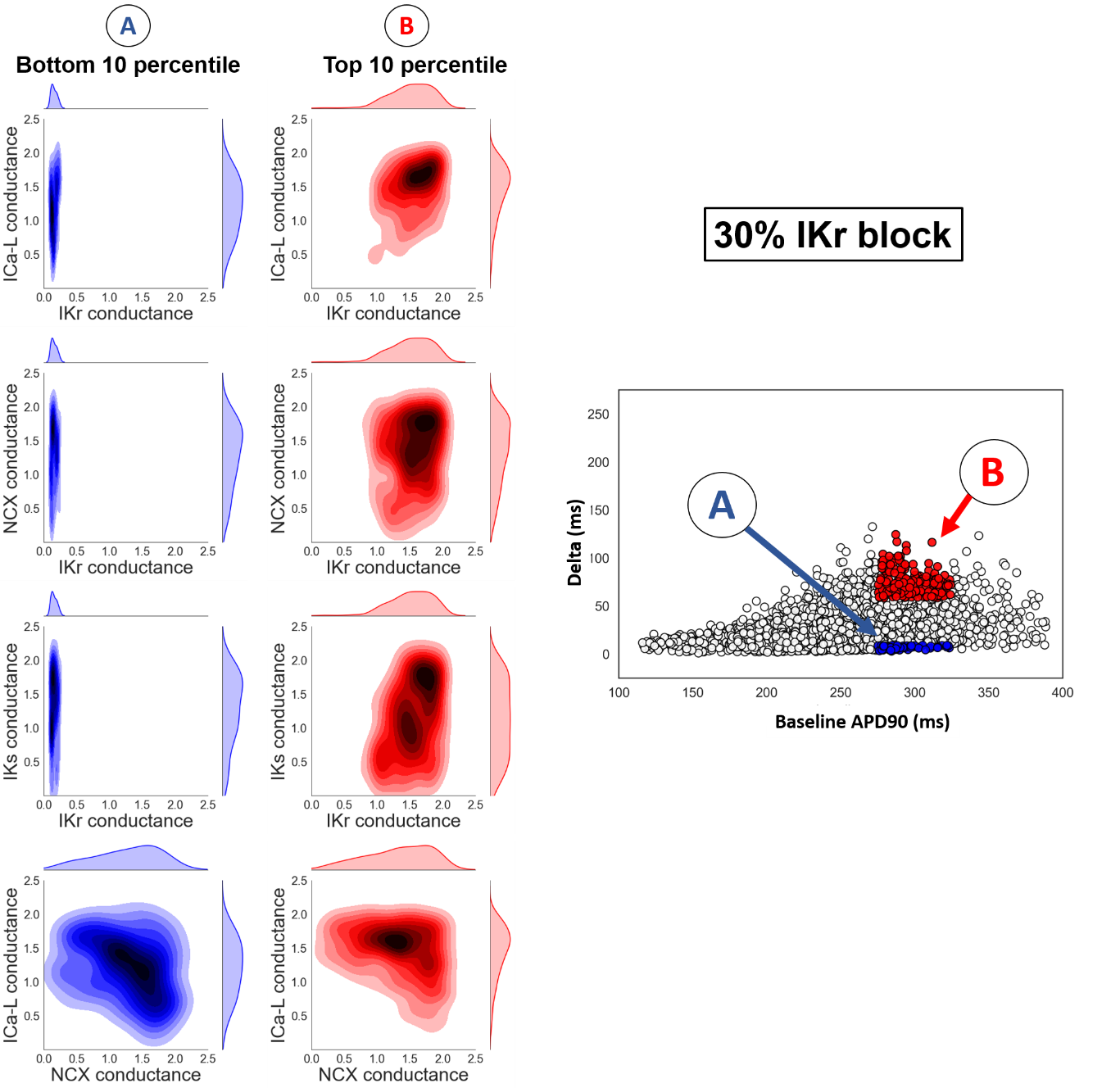


**Figure S7.** Kernel density estimation plots showing the relationship between selected ion channel pairs in two distinct responses to 30% IKr reduction. (A) low (<10%-ile) and (B) high (>90%-ile) responders to 30% IKr reduction. Action potentials in a narrow range (285-325ms) were selected at baseline to minimise APD-bias.

## Figure S8


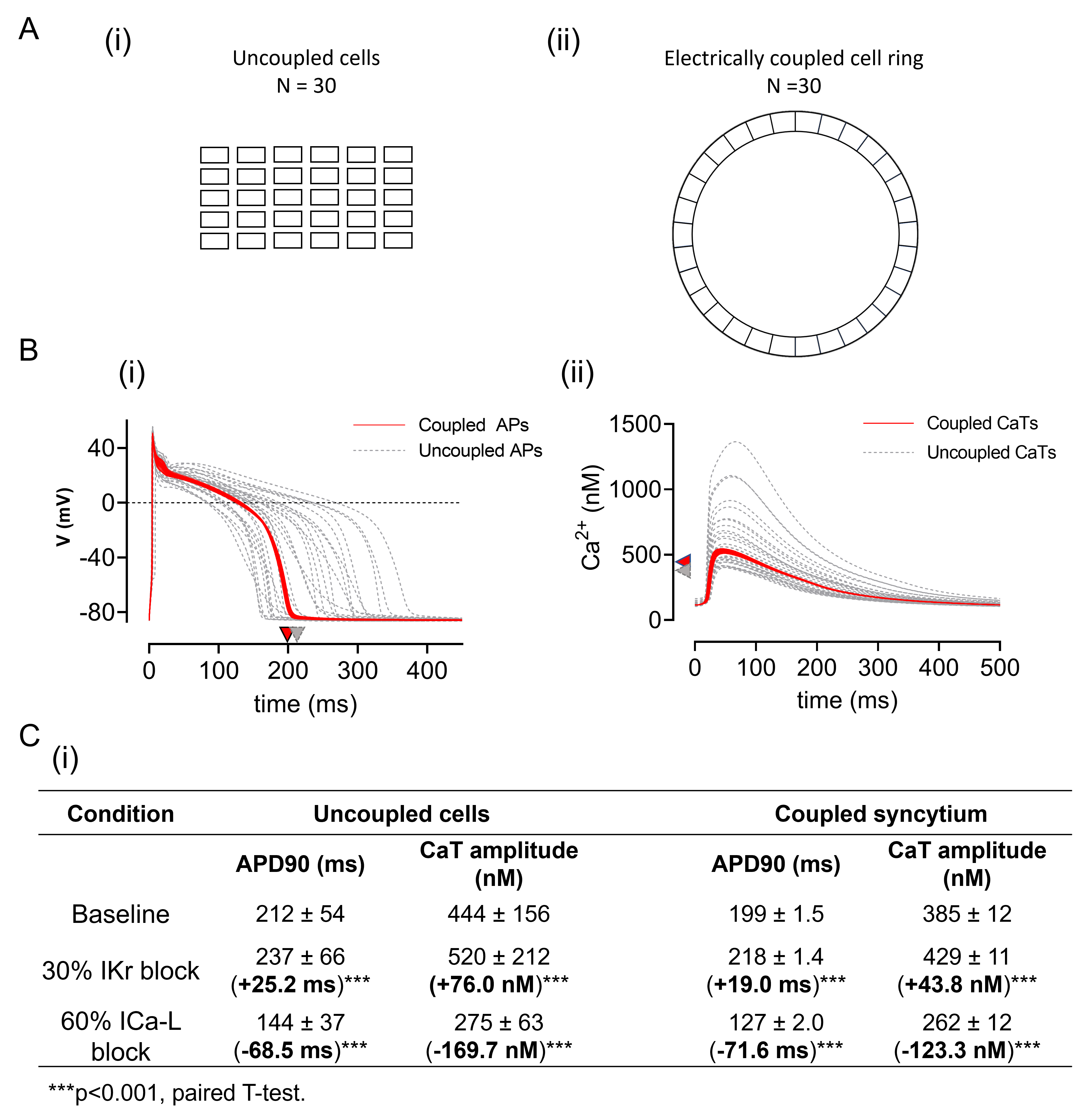


**Figure S8.** A(i, ii), diagrammatic representation of uncoupled and 1D coupled cellular models. B(i), uncoupled (grey dashed) and coupled (red) action potentials from 30 randomly sampled models from the normal response population. B(ii) uncoupled (grey dashed) and coupled (red) calcium transients of the models shown in B(i). C(i), summary table of averaged AP and calcium transient duration response to 30% IK(r) block, and 60% ICa(L) block, respectively.

## Figure S9


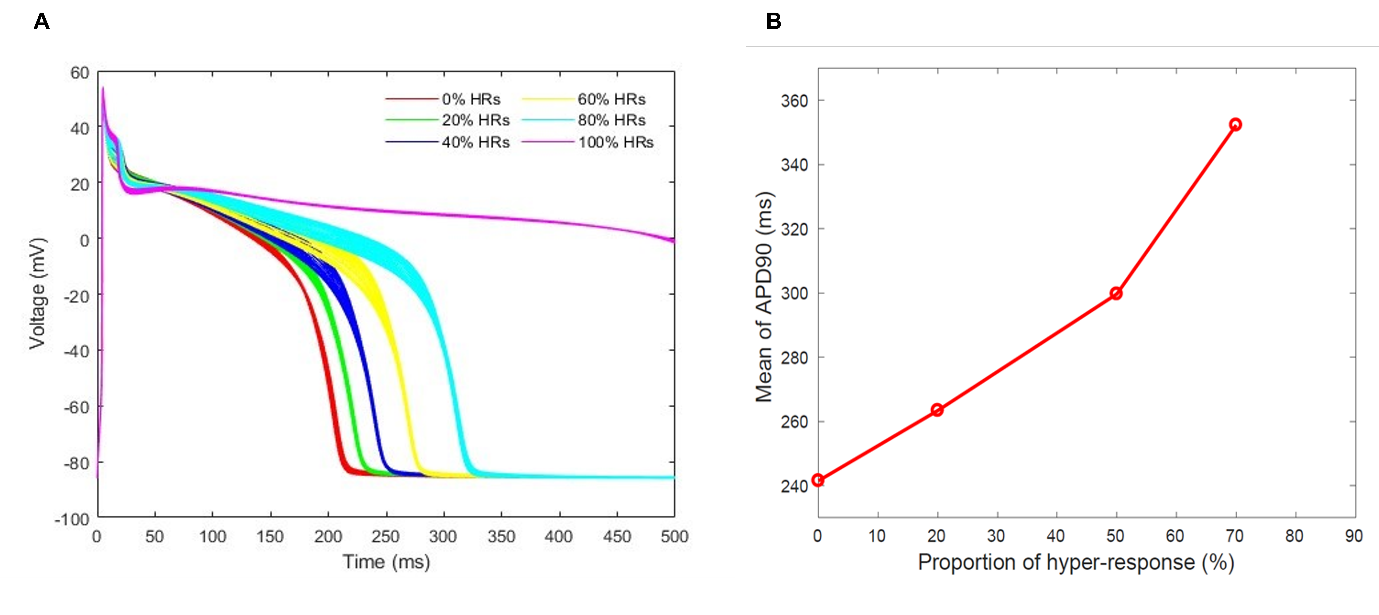
**Figure S9.** AP waveforms (n=30, respectively) generated from coupled ring with incremental proportions of hyper-responders (left). Mean APD_90_ of 30 models with incremental proportions of hyper-responders. The coupling strength was set to 0.6 mS/µF.

## Figure S10


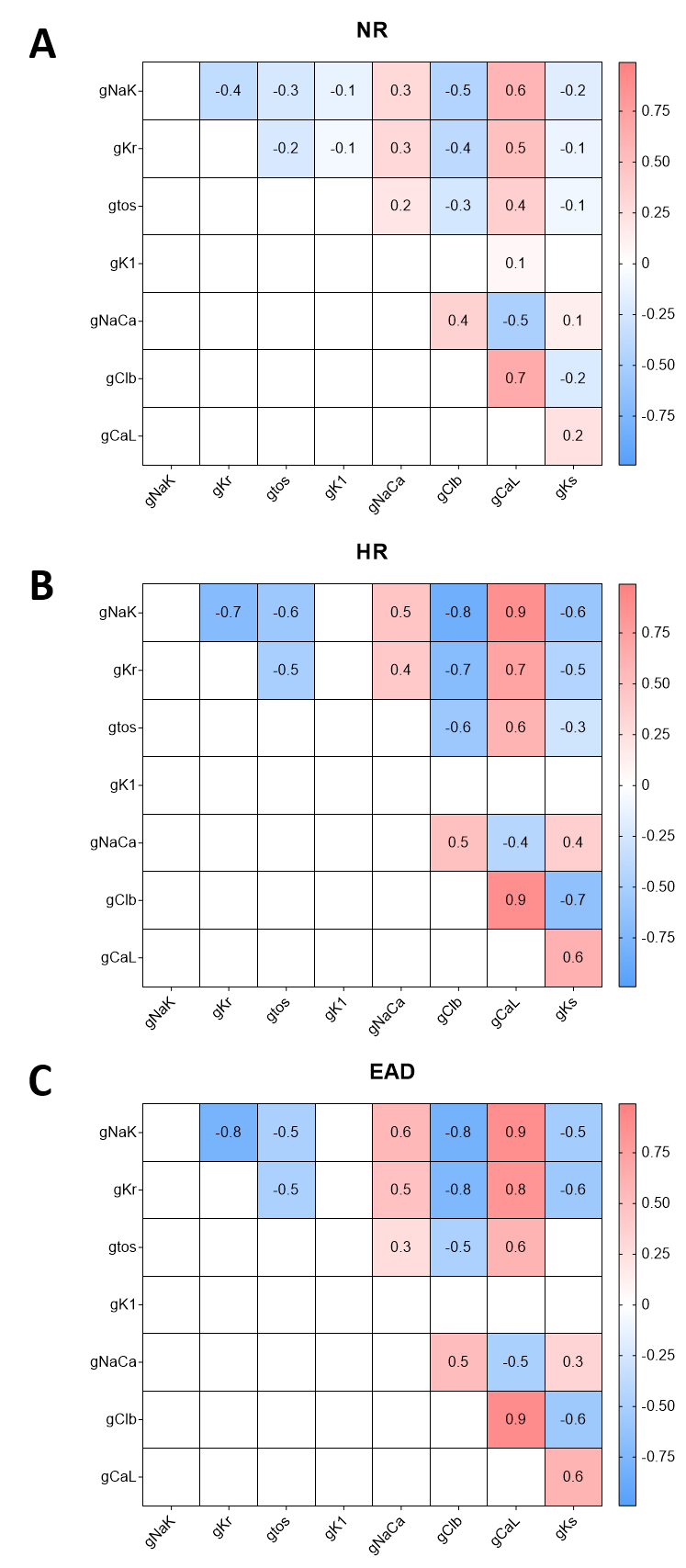


**Figure S10.** Partial correlation coefficient matrices of all channel conductance pairs. Non-significant (P>0.05) coefficients or those less than |0.1| are not shown. A, channel pair correlations in normal responders (NR); B, Channel pair correlations in hyper-responders (HRs) and C, channel pair correlations in models generating EADs. Value of correlation coefficient for each pair-wise comparison is shown in the appropriate square and colour coded according to the look-up table shown on the right.

## Figure S11


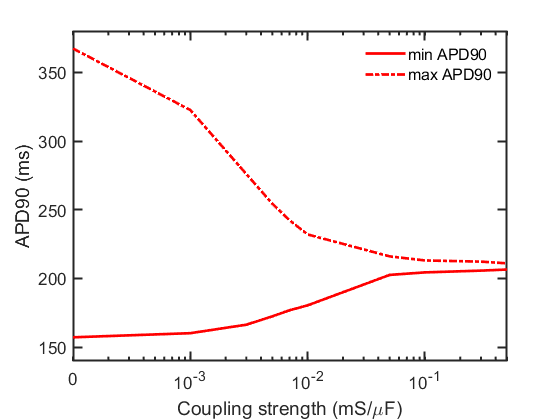


**Figure S11.** Change of APD90 range with increase of coupling strength (gap junctional conductance). Shown are the minimal and the maximal value of APD90 measured in a set of 30 cells drawn at random from the calibrated numerical single cell population and coupled in a ring as described in relation to Supplementary Figure S8.

## Figure S12


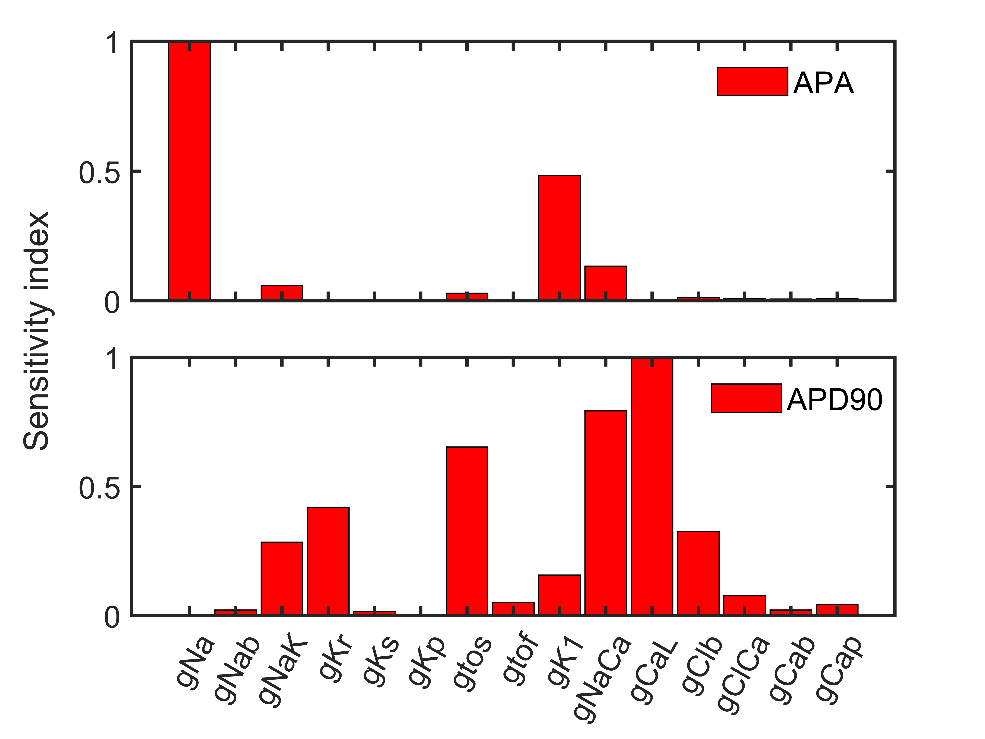


**Figure S12.** Sensitivity of selected AP biomarkers to variation of selected conductance values by 20% from the value of the published Shannon model. The computation of the sensitivity indices is detailed in the Supplementary Methods (below).

# Supplementary Methods

## Data analysis and trace filtering

Action potential trains of 2.5s were recorded, and all captured APs averaged (4-5 APs at 2Hz CL). All averaged waveforms were sampled at 10 kHz, and filtered using 15-point adjacent averaging. The upstroke time (TRise) was defined as time from 10% of upstroke to 90% of upstroke. APD_90_ was calculated as the duration in milliseconds from an artificial, simultaneous pulse generated upon cell stimulation, to 90% repolarisation.

## Computational sensitivity analysis and sensitivity indices

The sensitivity indices $S_{ij}$represent the normalized rate of change of a biomarker of interest $x_{i}$with respect to the variation of a single parameter of interest $p_{j}$, at all remaining parameters $p$ kept fixed at the “baseline” value $p_{0}$


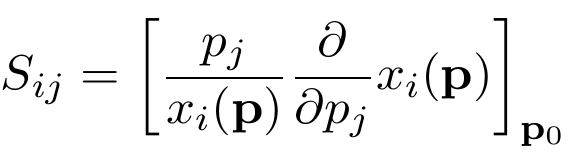


This expression is a conventional measure of local sensitivity (Snowden et al., 2017). The parameters values published in the original formulation of the Shannon model (17) are used as baseline. In Supplementary Figure S12 the values of the sensitivity indices are plotted for biomarkers APD90 and APA with respect to several selected  parameters as listed on the abscissa. The sensitivity indices are evaluated by using a first-order accurate forward difference formula for the discretisation of the given expression with discretisation step equal to 0.3 of the baseline values of the parameter being varied. Finally for ease of comparison, the values are normalised by S_APA,gNa_=0.191 (top panel) and S_APD90,gCaL_=0.490 (bottom panel), the largest in their respective sets.

## Stability of the Action Potential Train

Absence of instabilities cannot be guaranteed by comparing the last two of a train of APs. A guaranteed stable periodic response requires imposing periodic boundary value conditions for all dynamical variables at the beginning and the end of each AP, as detailed in (Simitev and Biktashev, 2011). However, such a boundary-value problem is difficult solve numerically without very good initial guess, which is not feasible to provide for each of the thousands of random model variations we consider. Instead, we have opted for a protocol where computational model variants are stimulated by a constant current pulse at 2Hz, a train of 1000 APs is computed, and model variants are rejected if the difference in APD90 between the last two APs was >5ms (3% of APD90). To further test for instability we have monitored plots akin to a Poincar’e map of the APD90 and the resting membrane potential and examples illustrating stable and unstable behaviour are included in the Supplementary Figure S13. This level of control is significantly stricter than that in experimental measurements and undetected instabilities are not expected to develop. The described protocol (a) has the advantage of being identical to our experimental protocol; and (b) is routinely used in similar numerical studies, e.g. (Kernik et al. 2019).

## Figure S13


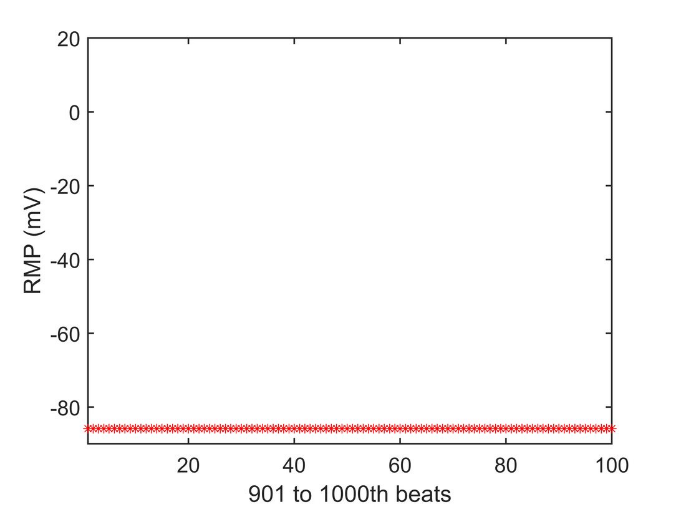

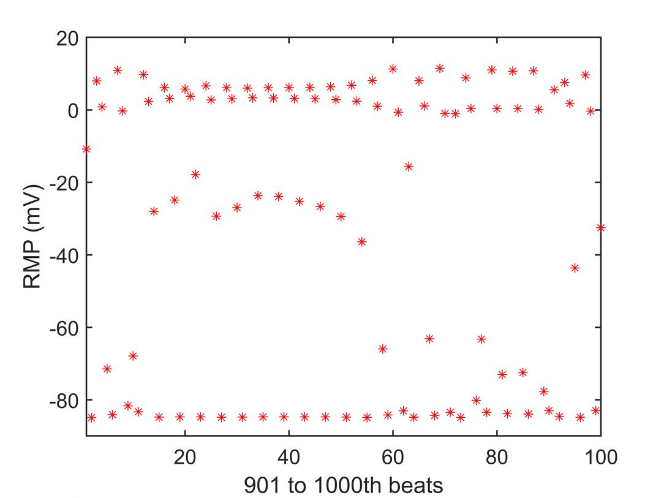


**Figure S13.**  Resting membrane potential measured at the end of the n-th AP. Examples of an abnormal response (left) and a normal response (right).

# Supplementary References

Kernik, D.C., Morotti, S., Wu, H., Garg, P., Duff, H.J., Kurokawa, J., Jalife, J., Wu, J.C., Grandi, E. and Clancy, C.E. (2019), A computational model of induced pluripotent stem-cell derived cardiomyocytes incorporating experimental variability from multiple data sources. J Physiol, 597: 4533-4564. https://doi.org/10.1113/JP277724

Simitev, R., Biktashev, V.N., Asymptotics of conduction velocity restitution in models of electrical excitation in the heart , Bull. Math. Biol., 73(1), pp. 72-115, 2011. doi.org/10.1007/s11538-010-9523-6.

Snowden, T. J., van der Graaf, P. H., & Tindall, M. J. (2017). Methods of Model Reduction for Large-Scale Biological Systems: A Survey of Current Methods and Trends. Bulletin of Mathematical Biology, 79(7), 1449–1486, doi:10.1007/s11538-017-0277-2].
